# Metformin alleviates bone loss in ovariectomized mice through inhibition of autophagy of osteoclast precursors mediated by E2F1

**DOI:** 10.1186/s12964-022-00966-5

**Published:** 2022-10-25

**Authors:** Xudong Xie, Liangcong Hu, Bobin Mi, Hang Xue, Yiqiang Hu, Adriana C. Panayi, Yori Endo, Lang Chen, Chenchen Yan, Ze Lin, Hui Li, Wu Zhou, Guohui Liu

**Affiliations:** 1 Department of Orthopedics, Union Hospital, Tongji Medical College, Huazhong University of Science and Technology, Wuhan, 430022 China; 2 Hubei Province Key Laboratory of Oral and Maxillofacial Development and Regeneration, Wuhan, 430022 China; 3 Division of Plastic Surgery, Brigham and Women’s Hospital, Harvard Medical School, Boston, MA 02215 USA

**Keywords:** Metformin, Osteoclast precursors, Bone loss, Autophagy, E2F1, BECN1, BNIP3

## Abstract

**Background:**

Postmenopausal bone loss, mainly caused by excessive bone resorption mediated by osteoclasts, has become a global public health burden. Metformin, a hypoglycemic drug, has been reported to have beneficial effects on maintaining bone health. However, the role and underlying mechanism of metformin in ovariectomized (OVX)-induced bone loss is still vague.

**Results:**

In this study, we demonstrated for the first time that metformin administration alleviated bone loss in postmenopausal women and ovariectomized mice, based on reduced bone resorption markers, increased bone mineral density (BMD) and improvement of bone microstructure. Then, osteoclast precursors administered metformin in vitro and in vivo were collected to examine the differentiation potential and autophagical level. The mechanism was investigated by infection with lentivirus-mediated BNIP3 or E2F1 overexpression. We observed a dramatical inhibition of autophagosome synthesis and osteoclast formation and activity. Treatment with RAPA, an autophagy activator, abrogated the metformin-mediated autophagy downregulation and inhibition of osteoclastogenesis. Additionally, overexpression of E2F1 demonstrated that reduction of OVX-upregulated autophagy mediated by metformin was E2F1 dependent. Mechanistically, metformin-mediated downregulation of E2F1 in ovariectomized mice could downregulate BECN1 and BNIP3 levels, which subsequently perturbed the binding of BECN1 to BCL2. Furthermore, the disconnect between BECN1 and BCL2 was shown by BNIP3 overexpression.

**Conclusion:**

In summary, we demonstrated the effect and underlying mechanism of metformin on OVX-induced bone loss, which could be, at least in part, ascribed to its role in downregulating autophagy during osteoclastogenesis via E2F1-dependent BECN1 and BCL2 downregulation, suggesting that metformin or E2F1 inhibitor is a potential agent against postmenopausal bone loss.

**Video abstract**

**Supplementary Information:**

The online version contains supplementary material available at 10.1186/s12964-022-00966-5.

## Background

Skeletal homeostasis regulation relies on a dynamic balance between osteoblasts-mediated bone formation and osteoclast-mediated bone resorption. Because of excessive osteoclastic activity, the balance of the process is disrupted and bone remodeling is imbalanced, leading to osteoporosis and other osteolytic conditions, such as postmenopausal osteoporosis [1, 2]. Therefore, inhibition of osteoclast formation and activation is a crucial therapeutic strategy for treating postmenopausal bone loss, such as the use of bisphosphonates, denosumab, and teriparatide. These drugs have been clinically ratified and show a favorable treatment effect in osteoporosis [3], but there are some adverse effects on other organs because of failure specifically target bone [4, 5]. Thus, novel drugs with low toxicity and/or more specific targets are necessary.

Osteoclasts originate from monocytes/macrophages of the hematopoietic lineage in bone marrow, which become multinucleated bone-resorbing osteoclasts via proliferation and differentiation [6]. Among them, macrophage colony-stimulating factor (M-CSF) and the receptor activator of nuclear factor-κB (RANK) ligand (RANKL) are regarded as crucial cytokines for formation of functional osteoclasts [1, 7, 8]. In addition to this canonical RANKL activation signaling pathway, autophagy, the major catabolic process of eukaryotic cells known to breakdown and recycle a wide array of cytoplasmic components [9, 10], actively participates in both the differentiation and bone-resorbing function of osteoclasts [11], in which the elevated level of autophagic activity is beneficial to osteoclasts formation and survival [12, 13]. Additionally, autophagy has also been demonstrated to be indispensable in the function of osteoclasts, in which the bone-resorbing activity is sharply reduced when osteoclast precursors were treated with bafilomycin, a specific inhibitor of vacuolar H^+^-ATPases and autophagy inhibitor. Inhibition of autophagy alleviates bone loss induced by glucocorticoid and ovariectomy [14]. Therefore, although different autophagy-inducing or intervening agents have different effects on the normal function and differentiation of osteoclasts, the previous basic and clinical studies have basically confirmed that autophagy affects the osteoclastsogenesis. Thus, autophagy inhibition may be an effective strategy for rescuing bone loss through downregulation of autophagy during osteoclastogenesis.

Metformin is the most commonly used hypoglycemic drug for patients with Type 2 diabetes mellitus (T2DM) because of its few side effects. Beyond that, it has also been proved that prior treatment with metformin is linked to reduced risk of osteoporosis in adult women without T2DM and obesity and fractures, particularly hip fractures [15, 16]. It has also been demonstrated that metformin plays a protective role in bone mass preservation under conditions of estrogen deficiency through an increase of osteoprotegerin (OPG) and decrease of RANKL expression [17]. In addition, studies indicated that metformin ameliorated arthritis through inhibition of osteoclastogenesis by suppressing the STAT3 and AMPK pathway and the expression of proinflammatory cytokines [18, 19]. However, current studies showed that metformin, as an autophagy modulator, is widely involved in various tissues protection, including the heart [20], kidney [21], and brain [22], but no such evidence has been proven yet for bone. Therefore, we hypothesized that metformin could inhibit osteoclast formation and activation by regulating autophagy during osteoclastogenesis in ovariectomized mice. Furthermore, deciphering the cellular and molecular mechanisms that metformin suppresses osteoclast activation is of seminal importance for understanding and treating bone loss and the development of osteoporosis. 

Current studies have shown that metformin could reduce osteoclast number and inhibit osteoclast activation, and prevent bone loss through suppression of RANKL signaling and AMPK/ NF-κb/ERK signaling pathway [17, 26, 27]. Additionally, in bone mesenchymal cells (BMSCs), strong experimental evidence indicates metformin is beneficial in bone formation and these effects may be partly attributed to activation of osteogenic differentiation by metformin via regulation of AMPK expression [23–25]. Thus, metformin is likely to be used for the prevention and treatment of bone loss when more information involving the effect of metformin in osteoclastogenesis becomes known. However, the premise is that the specific mechanism needs to be prior surfaced and proven.

## Materials and methods

### Reagents

Metformin was purchased from Sigma-Aldrich. The primary antibodies of GAPDH, BECN1, LC3, BNIP3, BCL2 and E2F1 were acquired from Proteintech (Wuhan, China). The 4, 6-diamidino-2-phenylindole (DAPI) was purchased from Solarbio (Beijing, China). The receptor activator of nuclear factor kappa-B ligand (RANKL) and macrophage-colony stimulating factor (M-CSF) were obtained from R&D Systems (Minnesota, USA). We purchased Minimum Essential Medium Alpha (MEM-α), fetal bovine serum (FBS), penicillin, streptomycin, and trypsin from Gibco (Grand Island, NY, USA). The cell counting kit-8 (CCK-8) was purchased from Dojindo (Kumamoto, Japan). Enzyme‐linked immunosorbent assay (ELISA) kits were acquired from CUSABIO (Wuhan, China). The TRAP staining kit was obtained from Solarbio (Beijing, China).

### Human peripheral blood samples and DXA examination

This study was carried out in full compliance with the Declaration of Helsinki, and authorized by Ethics Committee of Tongji Medical College, Huazhong University of Science and Technology, by approval number 2018 S431. All patients provided signed informed consent. Detailed information of the patients are listed in Table 1, and descriptive statistics for the obtained sample is listed in Table 2.

**Table 1:** Sample information included in this study

Sample	Gender	Age	Main condition	Drug	Dosage of metformin (g/d)	Treatment period of metformin(Year)	Years since diagnosis of T2DM	Years since menopause
1	F	57	2-DM	None	0	0	4.2	9.5
2	F	65	2-DM	Glimepiride	0	0	5.3	15.1
3	F	59	2-DM	None	0	0	3.8	5.6
4	F	57	2-DM	None	0	0	4.5	8.2
5	F	59	2-DM	None	0	0	5.1	10.5
6	F	60	2-DM	Xiaoke	0	0	4.1	11.7
7	F	62	2-DM	None	0	0	3.6	13.8
8	F	55	2-DM	None	0	0	4.6	6.1
9	F	58	2-DM	Metformin	2	2.3	4.8	8.9
10	F	64	2-DM	Metformin	2	1.8	3.5	15.8
11	F	65	2-DM	Metformin	2	2.5	3.8	14.6
12	F	55	2-DM	Metformin	2	2	4.6	5.9
13	F	59	2-DM	Metformin	2	1.9	5.1	9.8
14	F	57	2-DM	Metformin	2	1.6	3.4	7.4
15	F	63	2-DM	Metformin	2	2.4	6.2	13.5
16	F	64	2-DM	Metformin	2	2.1	5.3	15.4

F, Female; 2-DM, Type 2 diabetes mellitus

**Table 2:** Descriptive statistics for the obtained sample

	Mean age (Year)	Mean time after menopause (Year)	Mean time diagnosis of T2DM (Year)
Control group	59.3 ± 3.2	10.1 ± 3.4	4.4 ± 0.6
Metformin group	60.6 ± 3.8	11.4 ± 3.9	4.6 ± 1.0
*P*	0.45	0.47	0.6491

### Preparation of osteoclast precursors

Male C57BL/6 mice (5 weeks old) were purchased from the Center of Experimental Animals, Tongji Medical College, Huazhong University of Science and Technology. Osteoclast precursors (Bone marrow derived-macrophages, BMDMs) were obtained as previously described [28]. Briefly, bone marrow cells were harvested from mice femurs and tibias. After the red blood cells were lysed with RBC lysis buffer (Servicebio, Wuhan, China), the precipitated cells were cultured overnight in α-MEM medium (Gibco, Grand Island, NY, USA) with addition of 10% fetal bovine serum (Gibco) and 1% Penicillin–Streptomycin Solution (Gibco). Nonadherent cells were cultured in the presence of 30 ng/mL M-CSF for 5 d and differentiated into osteoclast precursors. Approximately 1 × 10^4^ osteoclast precursors were then added to a 96-well culture plate and differentiated into mature osteoclasts with 30 ng/mL M-CSF and 50 ng/mL RANKL.

### Cell Counting Kit 8 (CCK8)

For the CCK‐8 assay, approximately 1 × 10^4^ osteoclast precursors were added into 96‐well plates and cultured overnight. The 30 ng/mL M-CSF and 50 ng/mL RANKL were added to induce osteoclast differentiation with or without various concentrations of metformin for 5 d. The medium was replaced with serum-free medium containing CCK-8 reagent and incubated for 2 h, followed by detection of absorbance at 450 nm.

### Osteoclastogenesis assay in vitro

Approximately 1 × 10^4^ osteoclast precursors were added into each well of 96-well plate and cultured overnight. The medium was replaced with differentiation medium with or without metformin and incubated for 5 d. TRAP staining was carried out in accordance with the manufacturer’s instructions. TRAP-positive cells with greater than three nuclei were considered as mature osteoclasts. Osteoclastogenic ability was detected by quantification of the TRAP-positive area compared with the total area.

### Animal models and treatment

We used the ovariectomized (OVX) mice model in specific pathogen-free (SPF) facilities as previously described [29]. All animal studies were performed following protocols approved by the Laboratory Animal Center, Tongji Medical College, Huazhong University of Science and Technology and were carried out as regulated by the Tongji Medical College Animal Care and Use Committee. Eight-week-old female C57BL/6 mice were randomly distributed into three groups: sham group (served as controls), model group (mice subjected to bilateral OVX and treated with vehicle), and treatment group (mice underwent bilateral OVX and treated with metformin or E2F1-siRNA).

Briefly, mice were weighed and anesthetized with 1% pentobarbital by intraperitoneal injection, and then subjected to bilateral OVX or a sham operation. Four weeks later, mice were treated with metformin intraperitoneally (100 mg/kg) in the treatment group three times per week for 4 weeks. E2F1-RNAi (250 nmol/kg) was administrated through tail vein to the mice in the treatment group twice per week for 4 weeks. Then, the blood samples were obtained and centrifuged for 5 min at 1000 xg and the supernatant was stored at -80 °C. The levels of β-CTX and TRACP-5b were examined using the Enzyme‐linked immunosorbent Assay (ELISA). The mouse femurs were preserved for further analysis.

### Western blotting

Osteoclast precursors (4 × 10^5^ cells per well) were seeded into six-well plates and cultured overnight. After metformin treatment, total protein was extracted using a RIPA lysis buffer (50 mM Tris, 150 mM NaCl, 1% NP-40 and 0.5% sodium deoxycholate) with a proteinase inhibitor cocktail. Next, 10 µg protein was analyzed on 10% or 12.5% SDS-PAGE and electrophoretically transferred onto polyvinylidene fluoride (PVDF) membranes. After blocking with nonfat milk for 2 h, the PVDF membranes were incubated with specific antibodies at 4 °C overnight. Next, the membranes were washed three times for 10 min with TBS-T and incubated with anti-rabbit secondary antibodies for 1 h at room temperature. After three washes with TBS-T, the membranes were incubated in chemiluminescent substrate and visualized using the GeneGnome HR Image Capture System. The original bands are presented in Additional file 1.

### Co‐immunoprecipitation (Co‐IP)

Osteoclast precursors were treated with or without metformin in the presence of M-CSF and RANKL. The IP lysis buffer added to PMSF protease inhibitor and phosphatase inhibitor Cocktail (Proteintech; Wuhan, China) were used to lyse the cells for 30 min and precipitates were collected by centrifugation (12,000 rpm). Then 100 μg of total protein was incubated with anti‐BECN1 (5 μg) or anti‐BCL2 (5 μg) or anti-BNIP3 (5 μg) or non‐specific IgG antibodies overnight at 4 ℃ on a rocker. Among them, total protein treated with non‐specific IgG antibody served as a negative control. Then, the immunocomplexes were collected with 20 μl of protein A/G‐sepharose beads (Proteintech) and the proteins were denatured by boiling in SDS loading buffer for 10 min. Finally, proteins were separated on a 10% SDS-PAGE gel. The original bands are presented in Additional file 1.

### Immunofluorescence staining

Approximately 1 × 10^4^ osteoclast precursors were seeded into each well of a 96-well plate and cultured overnight. Cells were treated with metformin for 24 h in the presence of 30 ng/mL M-CSF and 50 ng/mL RANKL. After treatment, cells were kept in 4% paraformaldehyde (PFA) for 20 min, permeabilized with 0.5% Triton X‐100 for 10 min and then blocked with 5% goat serum for 30 min. After washing three times using PBS, the fixed cells were incubated with the primary antibodies (LC3, diluted 1:100) at 4 °C overnight, followed by DAPI staining for 5 min. Finally, the cells were observed using a digital microscope system (IX81; Olympus, Japan).

### Pit formation assay

Approximately 1 × 10^4^ osteoclast precursors were seeded into each well of a 96-well plate and cultured overnight. Then, cells were incubated with 30 ng/ml M-CSF and 50 ng/ml RANKL and the medium was changed every 2 days. After 5 days, osteoclasts were digested with collagenase and seeded into a Corning® Osteo Assay Surface 96-well Plate, and cultured for 4 days with metformin in the presence of 30 ng/ml M-CSF and 50 ng/ml RANKL. The culture medium was replaced and the surface was washed with 0.5% sodium hypochlorite. The plate was washed twice with PBS and left to dry at room temperature for 3 h. Finally, the resorbing area was visualized using a digital microscope system and analyzed with the ImageJ software.

### Enzyme‐linked immunosorbent assay (ELISA)

Blood supernatants were collected and then the levels of β-CTX and TRACP-5b were measured using an ELISA kit (CUSABIO, CSB-E08490h, CSB-E08492m, CSB-E11224h, CSB-E12782m, Wuhan, China) according to the instruction manual.

### Micro-CT analysis

Micro-computed tomography (micro-CT) (Bruker SkyScan 1176 scanner mCT system) was used to analyze the femur structure. Under the analysis conditions with 37 kV and 121 mA, 300 section planes were examined from the growth plate. To perform the morphometric analysis, we obtained the following trabecular parameters, BMD, BV/TV (%), BS/TV, and Tb.N, from the software of CTan and CTvol.

### Hematoxylin and Eosin (H&E) Staining and TRAP Staining

The femurs were collected from mice and fixed in 4% paraformaldehyde for 4 d. The samples were subsequently decalcified for 2 weeks using 10% tetracycline-EDTA (Servicebio) and 4-μm-thick paraffin-embedded sections were prepared with H&E and TRAP staining for further analysis. H&E and TRAP staining were carried out according to the manufacturers’ instructions (Solarbio). The images were obtained by microscopy and histological analyses were performed using ImageJ software.

### Overexpression lentivirus transfection

Osteoclast precursors were seeded into six well plates at 4 × 10^5^ cells/well. The cells were transfected with lentiviral particles for overexpression of BNIP3 or E2F1 (Genechem, Shanghai, China) using polybrene in the presence of 30 ng/mL M-CSF. After 48 h and washing three times to stop the infection, cells were incubated with vehicle or metformin in the presence of 30 ng/mL M‐CSF and 50 ng/mL RANKL.

### In vivo E2F1-siRNA

E2F1-siRNA was purchased from RIBOBIO (Guangzhou, China). The E2F1-siRNA targeting sequence was 5'-GACGTGTCAGGACCTTCGTT-3'. To enhance stability in serums and transfection efficiency, cholesterol and methylation-modified siRNA were performed.

For siRNA-mediated gene knockdown in vivo, E2F1-siRNA was administrated by tail vein to the ovariectomized mice twice per week for 4 weeks, and then the knockdown efficiency was evaluated.

### Autophagy monitoring

Osteoclast precursors were infected with tandem GFP-red fluorescent protein (RFP)-LC3 lentivirus (Genechem) for 16–18 h according to the manufacturer’s instructions. After 3 d, cells were treated with metformin or/and rapamycin (RAPA, 10 nM) for 24 h to observe the autophagy flux. When autophagy was induced, the overlap between GFP and RFP was shown as yellow dots representing autophagosomes. When autophagosomes fuse with lysosomes and form autolysosomes, the GFP degrades in the acidic environment, but RFP-LC3 remains evident as red dots.

### Statistical analysis

All experiments were conducted at least three times and are presented as the mean ± standard deviation. One-way analysis of variance for three groups and Student’s t-test for two groups were performed using GraphPad Prism 8.0 (GraphPad Software). A value of p < 0.05 was considered statistically significant.

## Results

### Change in bone resorption and BMD with metformin in postmenopausal women with T2DM

**Fig. 1:**
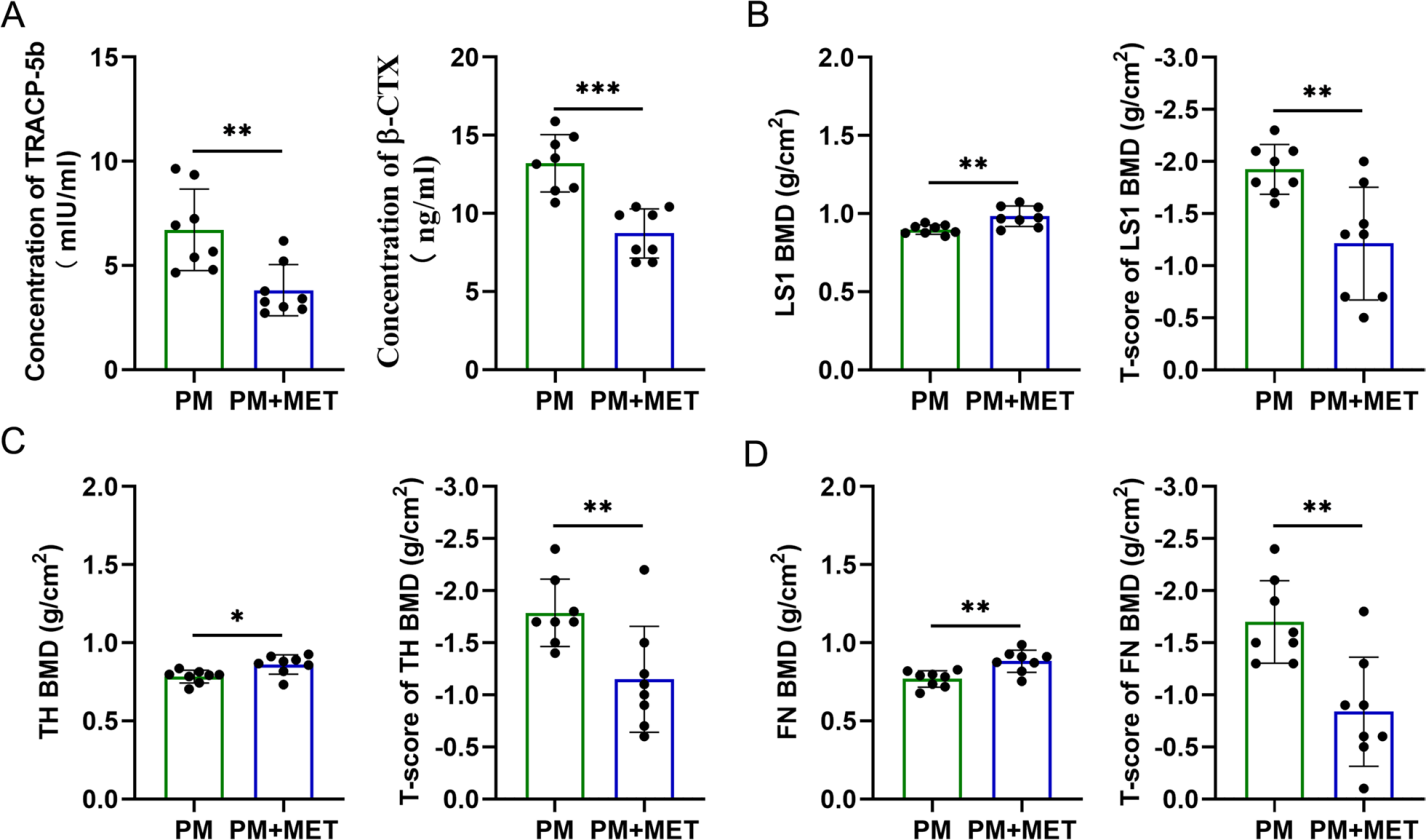
Change in bone resorption and BMD with metformin in postmenopausal women with T2DM. **A** The β-CTX and TRACP-5b levels in the serum of postmenopausal women taking or not taking metformin for T2DM. **B** BMD and BMD T-score at LS1; **C** BMD and BMD T-score at TH. **D** BMD and BMD T-score at FN. PM, Postmenopausal women (n = 8); PM + MET, metformin-treated postmenopausal women(n = 8); LS1, Lumbar Spine; TH, Total Hip; FN, Femoral Neck. Data are presented as means ± SD of 8 independent experiments (*p < 0.05; **p < 0.01; ***p < 0.001)

In order to investigate whether metformin contributes to bone health, we carried out a study using blood samples from 16 postmenopausal women taking or not taking metformin for T2DM, and then we determined the levels of β-CTX and TRACP-5b in the serum, reflecting osteoclast activity and bone resorption. The results showed that the levels of β-CTX and TRACP-5b in the serum were markedly decreased in patients with metformin treatment (Fig. 1A). In addition, these patients agreed to undergo testing of dual-energy x-ray absorptiometry (DXA), and we found that treatment with metformin led to comparable results in bone mineral density (BMD) at lumbar spine 1 (LS1) (Fig. 1B), total hip (TH) (Fig. 1C), and femoral neck (FN) (Fig. 1D). Accordingly, the mean BMD T-score at LS1, TH and FN showed significant increases in response to metformin treatment (Fig. 1B–D).

### Metformin attenuates OVX-induced bone loss in vivo

**Fig. 2:**
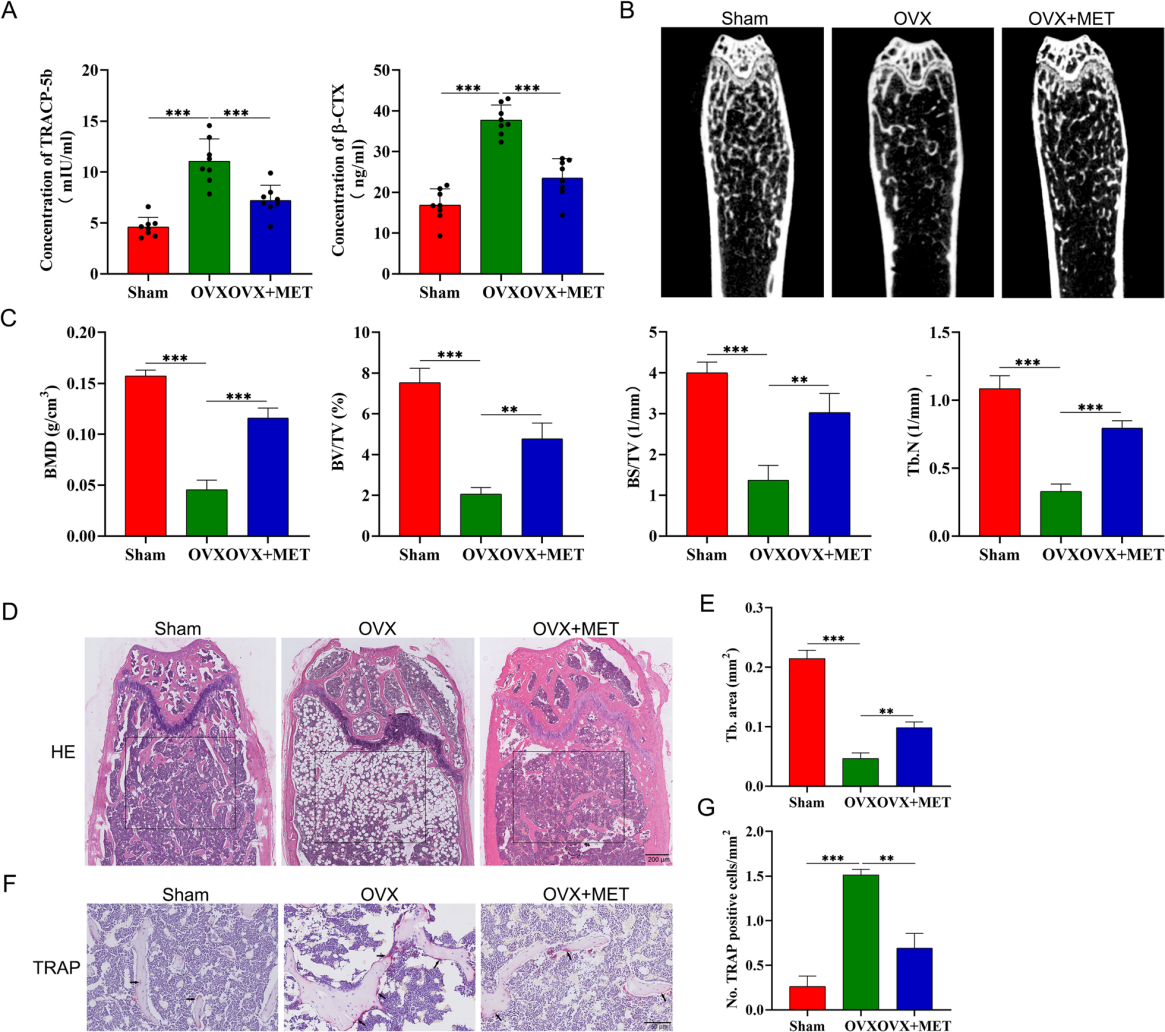
Metformin attenuates OVX-induced bone loss in vivo. **A** The β-CTX and TRACP-5b levels in the serum from sham group (n = 8), ovariectomy (OVX; n = 8) group and OVX + MET group (n = 8). **B** Representative micro-CT images of the distal femur in sham group, OVX group, and OVX + MET group. **C** Quantitative analysis of bone mineral density (BMD), trabecular bone surface area/total value (BS/TV), volume per tissue volume (BV/TV) and trabecular number (Tb. N). **D** Representative images of Hematoxylin and Eosin staining of distal femur sections. **E** Quantitative analysis of trabecular (Tb) bone area in (D). **F** Representative images of femur stained with TRAP. **G** Histomorphometric analysis of the osteoclast number. sham, sham operated mice group; OVX, ovariectomized mice group, OVX+MET, ovariectomized mice with metformin treatment group. (*p < 0.05; **p < 0.01; ***p < 0.001)

To prove the protective effect of metformin against OVX-induced bone loss in vivo, blood samples were collected from ovariectomized mice that had or had not received metformin. Metformin treatment for 4 weeks in ovariectomized mice resulted in lower concentration of the circulating bone resorption markers, including β-CTX and TRACP-5b, compared with vehicle-treated ovariectomized mice (Fig. 2A). To further explore the bone microstructural changes after metformin treatment, microcomputed tomography (micro-CT) was used to evaluate various indicators in the distal femur, including BMD, bone volume/total volume (BV/TV), trabecular bone surface area/total value (BS/TV), and trabecular number (Tb. N). The results highlighted reduced bone mass (BMD and BV/TV) and deteriorated trabecular architecture (Tb. N) in the ovariectomized mice, and treatment with metformin attenuated this loss of bone and bone quality (Fig. 2B, C). In addition, hematoxylin and eosin staining showed that the ovariectomized mice had trabecular bone loss compared with the sham-operated mice, whereas bone loss was reduced in ovariectomized mice administered with metformin (Fig. 2D, E). It is well known that osteoporosis after menopause is often attributed to increased osteoclastic bone resorption caused by estrogen deficiency [30]. We therefore investigated the effects of metformin on osteoclast formation and carried out TRAP staining to investigate the distribution of osteoclasts in the femur. As shown in Fig. 2F, G, the number of osteoclasts was significantly increased in ovariectomized mice, while metformin administration significantly reduced the number of osteoclasts.

**Fig. 3:**
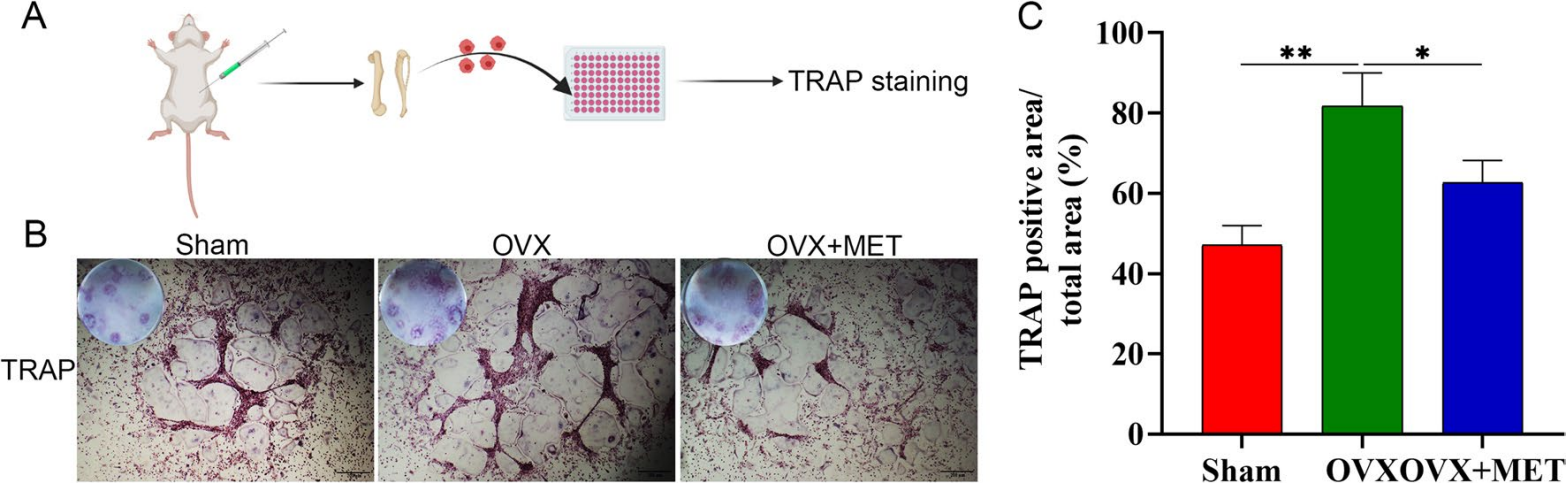
Metformin in vivo administration inhibits the formation of osteoclasts. **A** schematic diagram of the experiment. **B**, **C** TRAP-staining and quantitation of TRAP-positive cells. Data are presented as means ± SD of 3 independent experiments (*p < 0.05; **p < 0.01; ***p < 0.001)

Studies have indicated that metformin suppressed osteoclast formation potentially through stimulating OPG and reducing RANKL expression in vivo [17]. To further test whether other mechanisms may be involved, we examined the differentiation potential of osteoclast precursors isolated from sham mice, ovariectomized mice, and ovariectomized mice treated with metformin with the addition of equal amounts of RANKL in vitro (Fig. 3A). Osteoclast precursors were stimulated with 30 ng/mL M-CSF and 50 ng/mL RANKL for 5 d, and their differentiation into mature osteoclasts was assessed by TRAP staining. In a manner consistent with our observation of a decrease in bone loss, we found fewer TRAP-positive osteoclasts along with smaller osteoclasts in ovariectomized mice administered with metformin, as compared to those in ovariectomized mice (Fig. 3B, C). Taken together, these data suggest that metformin has protective effect against OVX-induced bone loss in vivo at least partially through inhibition of osteoclast formation and activity via other mechanisms in addition to OPG/RANKL.

### Metformin suppresses RANKL-induced osteoclast differentiation in vitro

**Fig. 4:**
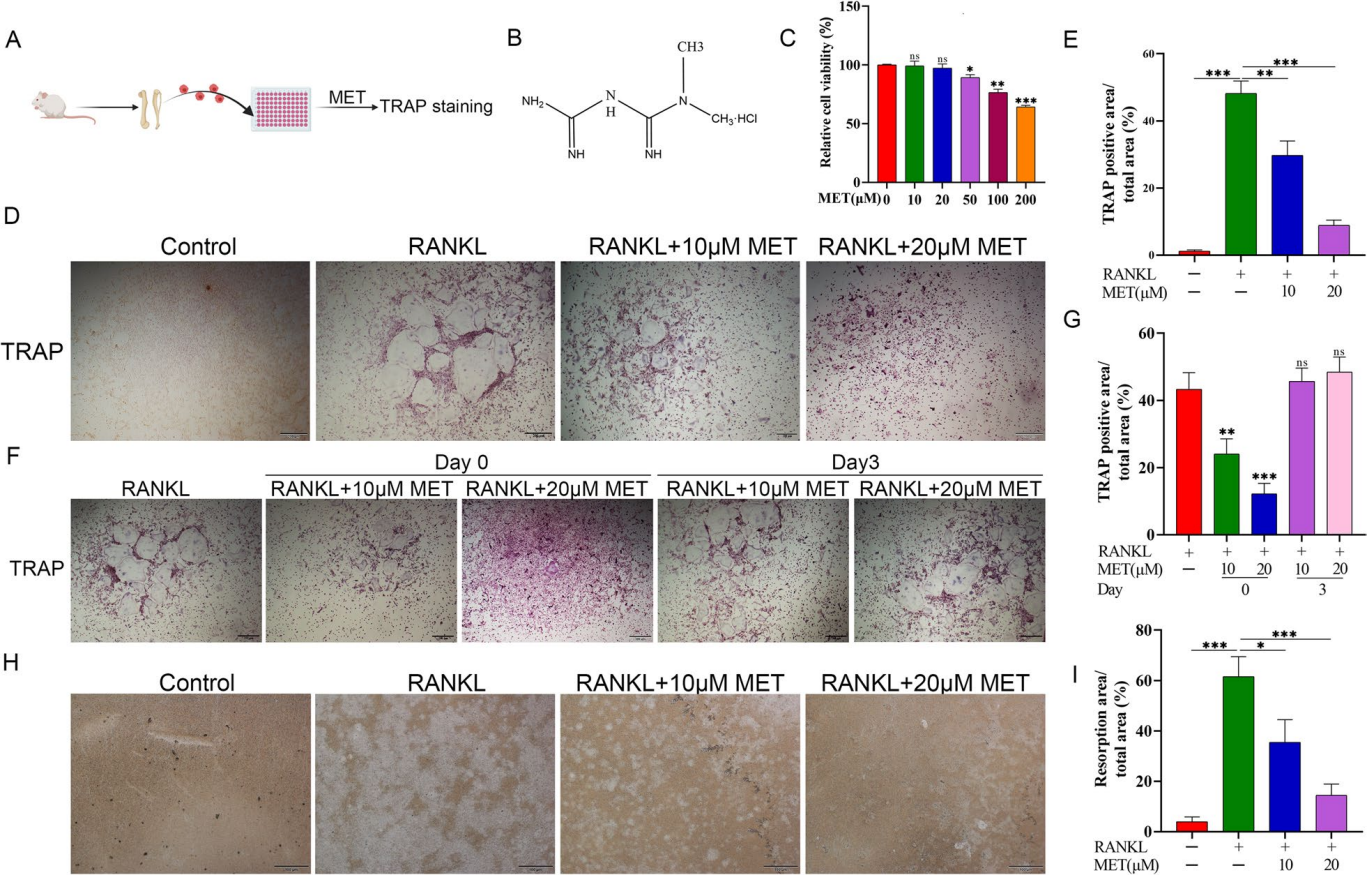
Metformin suppresses RANKL-induced osteoclast differentiation in vitro. **A** schematic diagram of the experiment. **B** Chemical structural formula of metformin. **C** Osteoclast precursors were incubated with different concentrations of metformin for 5 d in the presence of 30 ng/ml M-CSF and 50 ng/mL RANKL. Subsequently, cell viability was evaluated with Cell Counting Kit 8 (CCK8) reagent. **D** Representative images of TRAP-positive cells after treatment with metformin. **E** Quantitative analysis of the area of TRAP-positive osteoclasts. **F**, **G** Effect of metformin on RANKL-induced osteoclast precursors differentiation at different stages. **H**, **I** Pit formation assay of osteoclasts and quantification of the pits area. Data are presented as means ± SD of 3 independent experiments (*p < 0.05; **p < 0.01; ***p < 0.001)

We next investigated metformin in osteoclast differentiation in vitro. To directly test this, osteoclast precursors isolated from 5-week-old male C57BL/6 mice were stimulated with or without metformin in the absence/presence of RANKL, and then osteoclastogenesis was assessed by TRAP staining (Fig. 4A). Before in vitro studies, the cell counting kit 8 (CCK8) analysis was performed to determine the appropriate concentration of metformin (Fig. 4B). As shown in Fig. 3C, metformin below 20 μM had nearly no cytotoxic effects on the osteoclast precursors. Furthermore, RANKL treatment resulted in notably greater osteoclast number and size, whereas an addition of metformin in the osteoclast precursors significantly reduced the number of TRAP-positive cells in a dose-dependent manner (Fig. 4D, E). To confirm the effects of metformin on RANKL-induced osteoclast precursors differentiation into mature osteoclasts, metformin was added to osteoclasts differentiation cultures beginning on d 0 and d 3 for osteoclast precursors. The results indicated that metformin inhibited osteoclastogenesis on the first day but had little effect on osteoclastogenesis at later stages (Fig. 4F,G). However, a high concentration of metformin may have an effect on osteoclast differentiation at later stages via promoting osteoclast precursors or osteoclasts apoptosis [31], which needs further verification. Furthermore, to verify the direct effect of metformin on the bone resorption activity of mature osteoclasts, pit formation assay was performed. The results indicated that treatment with metformin could significantly decreased the bone resorption area compared with vehicle treatment (Fig. 4H, I). In summary, metformin could directly inhibit RANKL-induced osteoclast differentiation and activity in vitro.

### Metformin inhibits osteoclast differentiation via decreasing autophagy of osteoclast precursors

**Fig. 5:**
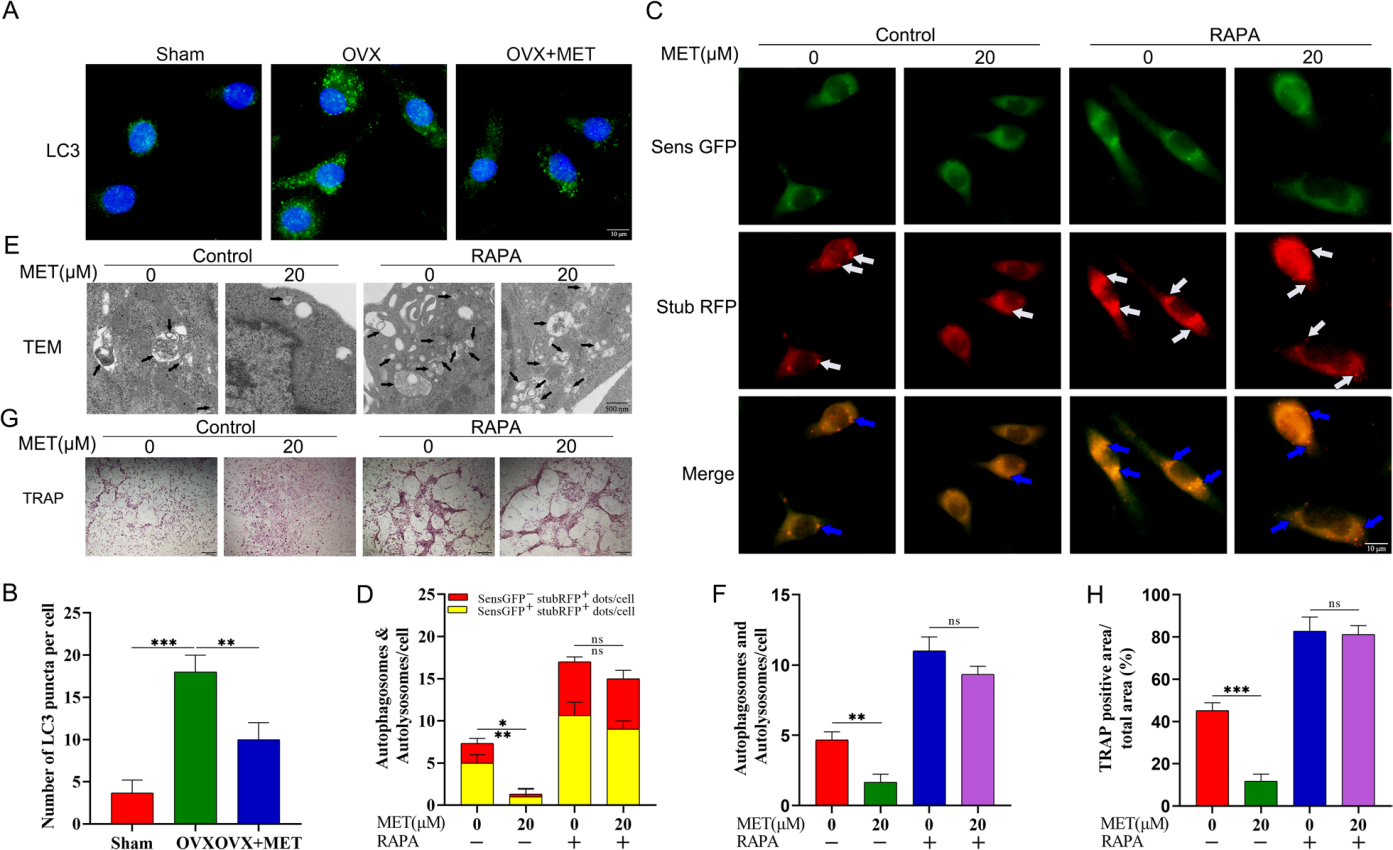
Metformin inhibits osteoclast differentiation via decreasing autophagy. **A** Immunofluorescence staining for LC3 in osteoclast precursors isolated from the femur and tibia of sham group mice, OVX group mice, and OVX + MET group mice. **B** Quantification of the LC3 puncta per cell in (**A**). **C**, **D** After infection with tandem GFP-red fluorescent protein (RFP)-LC3 lentivirus, osteoclast precursors were treated with metformin and/or rapamycin (10 nM) for 24 h in presence of 30 ng/mL M-CSF and 50 ng/mL RANKL, and then the fluorescence was observed by fluorescence microscopy and quantitative analysis was performed. **E**, **F** metformin and/or rapamycin were used to treat osteoclast precursors for 24 h, and then the formation of autophagosomes and/or autolysosomes in osteoclast precursors were observed under transmission electron microscopy. **G** After metformin or/and rapamycin, TRAP-stained multinucleated osteoclasts. **H** Quantitative analysis of the area of TRAP-positive osteoclasts. Data are presented as means ± SD of 3 independent experiments (*p < 0.05; **p < 0.01; ***p < 0.001, ns, non-significant)

There are increasing findings demonstrating that, in addition to the interrelation between autophagy and bone physiology, autophagy plays a crucial role in the onset and progression of pathological osteoporosis [32, 33]. Moreover, downregulation of autophagy inhibits ovariectomy-induced bone loss [14]. In the present study, we found obvious accumulation of LC3 puncta in osteoclast precursors isolated from ovariectomized mice as compared with the corresponding control. However, metformin-treated ovariectomized mice manifested significantly fewer LC3 puncta compared with the vehicle-treated group (Fig. 5A, B).

To further confirm these data, we used tandem GFP-red fluorescent protein (RFP)-LC3 lentivirus to infect osteoclast precursors for labeling and tracking LC3. Red puncta indicate autophagolysosomes, and yellow puncta overlapping red and green fluorescence are autophagosomes. The count of red and yellow puncta could be used to infer the intensity of autophagy flow [34]. The results shown in Fig. 5C demonstrate that metformin could dramatically lessen the intensity of autophagy flux during RANKL-induced osteoclast differentiation, in which the white arrows represent autophagolysosomes and the blue arrows represent autophagosomes, then quantification of the number of autophagolysosomes (red) and autophagosomes (yellow) in Fig. 5C was shown in Fig. 5D, which remained consistent. Electron microscopy imaging results were in line with this, suggesting metformin could inhibit the formation of autophagosomes and/or autophagolysosomes during osteoclastogenesis (Fig. 5E, F). In order to expound the role of metformin-downregulated autophagy during osteoclast differentiation, rapamycin, a common autophagic activator, was used to activate metformin-downregulated autophagy. After introduction of rapamycin, metformin-reduced autophagy was reactivated, in which metformin could no longer fulfill its role in inhibiting autophagolysosomes or/and autophagolysosomes during osteoclastogenesis (Fig. 5C–F). Furthermore, rapamycin cotreatment blocked the ability to suppress osteoclast formation (Fig. 5G, H). Overall, metformin suppressed osteoclast differentiation most likely via downregulation of autophagy in osteoclast precursors, while rapamycin cotreatment could counteract these beneficial effects.

### Metformin regulates autophagy through a mechanism involving BECN1 and BNIP3

**Fig. 6:**
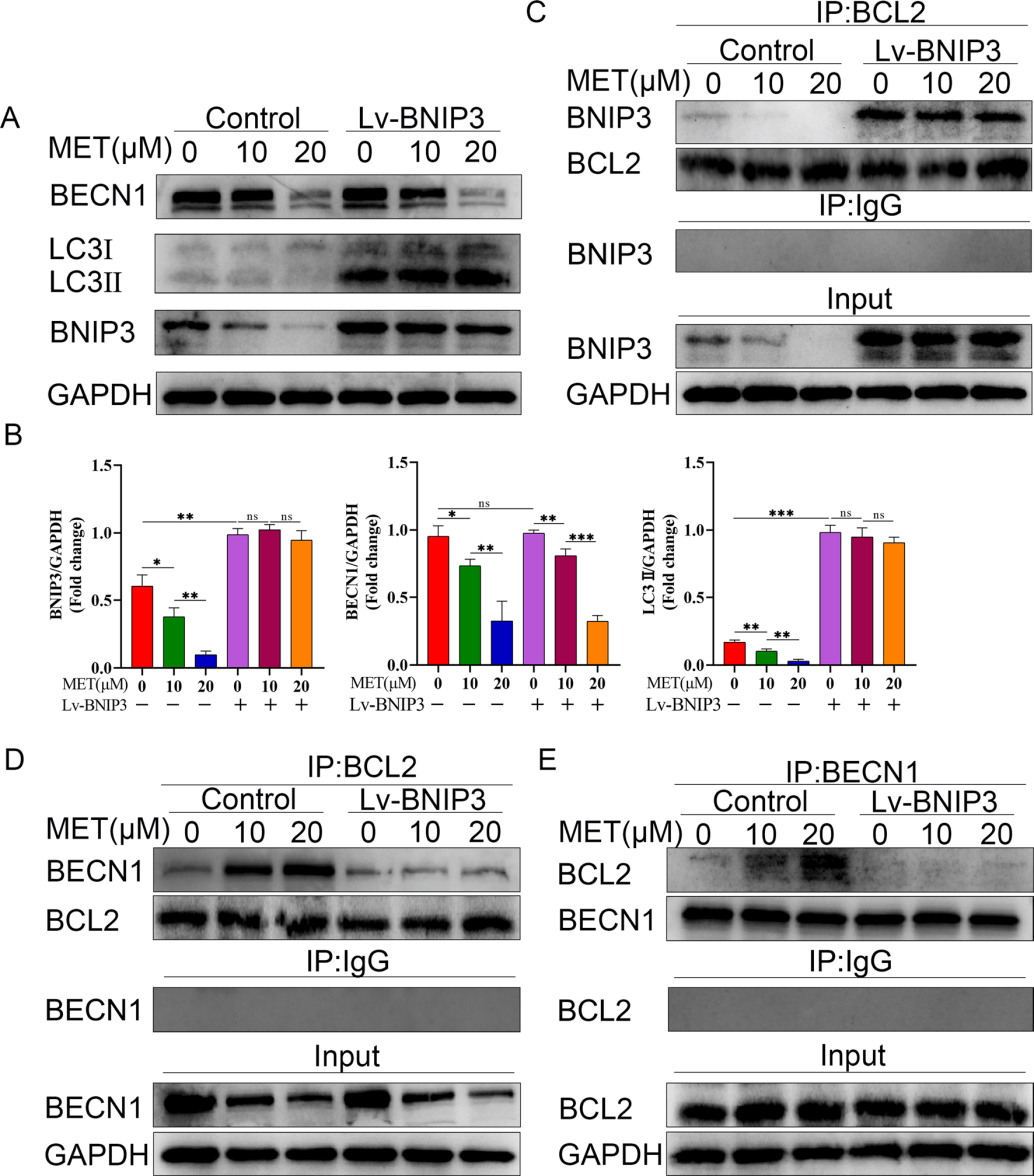
Metformin regulates autophagy through a mechanism involving BECN1 and BNIP3. **A** Cell lysates of osteoclast precursors were used to examine the expression of BECN1, BNIP3 and LC3 proteins by western bolt. After overexpression of BNIP3 (Lv-BNIP3), osteoclast precursors were treated with metformin (0, 10, 20 μM) for 5 d in the presence of 30 ng/mL M-CSF and 50 ng/mL RANKL. **B** The quantitative analysis of immunoblots relative to GAPDH protein level in (**A**). The results were from pairwise comparisons within- and between-group. **C** Osteoclast precursors were treated as in (**A**), and the cell lysates were preformed to co-immunoprecipitation with BCL2 antibody, followed by immunoblotting with the indicated antibodies. Among them, IgG group was considered as negative control. **D**, **E** Osteoclast precursors were treated as in (**A**), and the cell lysates were subjected to co-immunoprecipitation with either BCL2 or BECN1 antibody, followed by immunoblotting with the indicated antibodies. Among them, IgG group was considered as negative control. Values displayed are means ± SD of 3 independent experiments (*p < 0.05; **p < 0.01; ***p < 0.001, ns, non-significant)

After substantiating the cellular effects of metformin, we aimed to further explore the intrinsic molecular mechanism of metformin-downregulated autophagy. There have been numerous studies indicating that metformin regulates autophagy in an AMPK-dependent manner [35, 36]. However, the AMPK mechanism which was activated by metformin to enhance autophagy seems inconsistent with its effects on osteoclast differentiation. Thus, inhibitory effects on osteoclast differentiation caused by metformin may involve other mechanisms rather than those of the AMPK pathway. BECN1, as a highly evolutionarily conserved molecule of autophagy, is currently widely recognized for its role in autophagosome formation and bone homeostasis [37, 38] and can be regulated at several levels, including transcription, translation and post-translational modifications [39, 40]. Therefore, we subsequently analyzed whether the change of BECN1 expression participated in metformin-downregulated autophagy. As shown in Fig. 6A, B, treatment with metformin could downregulate the expression level of BECN1 protein in a dose-dependent manner.

Although the BCL2 family was initially considered to be important regulators of apoptosis [41], it is now increasingly recognized that it plays a crucial role in regulating not only apoptosis but also autophagy [42]. Thus, we next determined whether BCL2-family proteins participate in the mechanism of metformin-downregulated autophagy. As shown in Fig. 6A, B, we found a marked diminution in the expression level of the BH3-only protein, BNIP3, after metformin treatment compared with RANKL treatment only in a dose-dependent manner. Overexpression of BNIP3 by lentiviral transfection increased the LC3II protein expression, but did not affect the expression levels of BECN1 protein (Fig. 6A, B). These results indicated that metformin decreased autophagy of osteoclast precursors probably through a mechanism involving BECN1 and BNIP3. However, the downregulated level of BECN1 protein is independent from the level of BNIP3. Therefore, it is important to disentangle the relationship between BECN1 and BNIP3.

Recently, some studies revealed that BECN1 could interact with BCL2 to suppress autophagy [43, 44]. Given that BNIP3 is established as a BH3-only protein, it is most likely that metformin-mediated BNIP3 downregulation might result in an alteration in the connection between BECN1 and BCL2. In fact, co-immunoprecipitation revealed that metformin treatment lessened the binding between BNIP3 and BCL2 (Fig. 6C), followed by promoting the association between BECN1 and BCL2 and reduction of the free BECN1 level as compared with RANKL treatment alone (Fig. 6D, E). However, overexpression of BNIP3 abolished the metformin-mediated BNIP3 diminishment, and promoted the BNIP3 and BCL2 interaction, leading to disassociation between BCL2 and BECN1 (Fig. 6C–E).

### Metformin-mediated autophagy downregulation via BECN1 and BNIP3 is E2F1 dependent

**Fig. 7:**
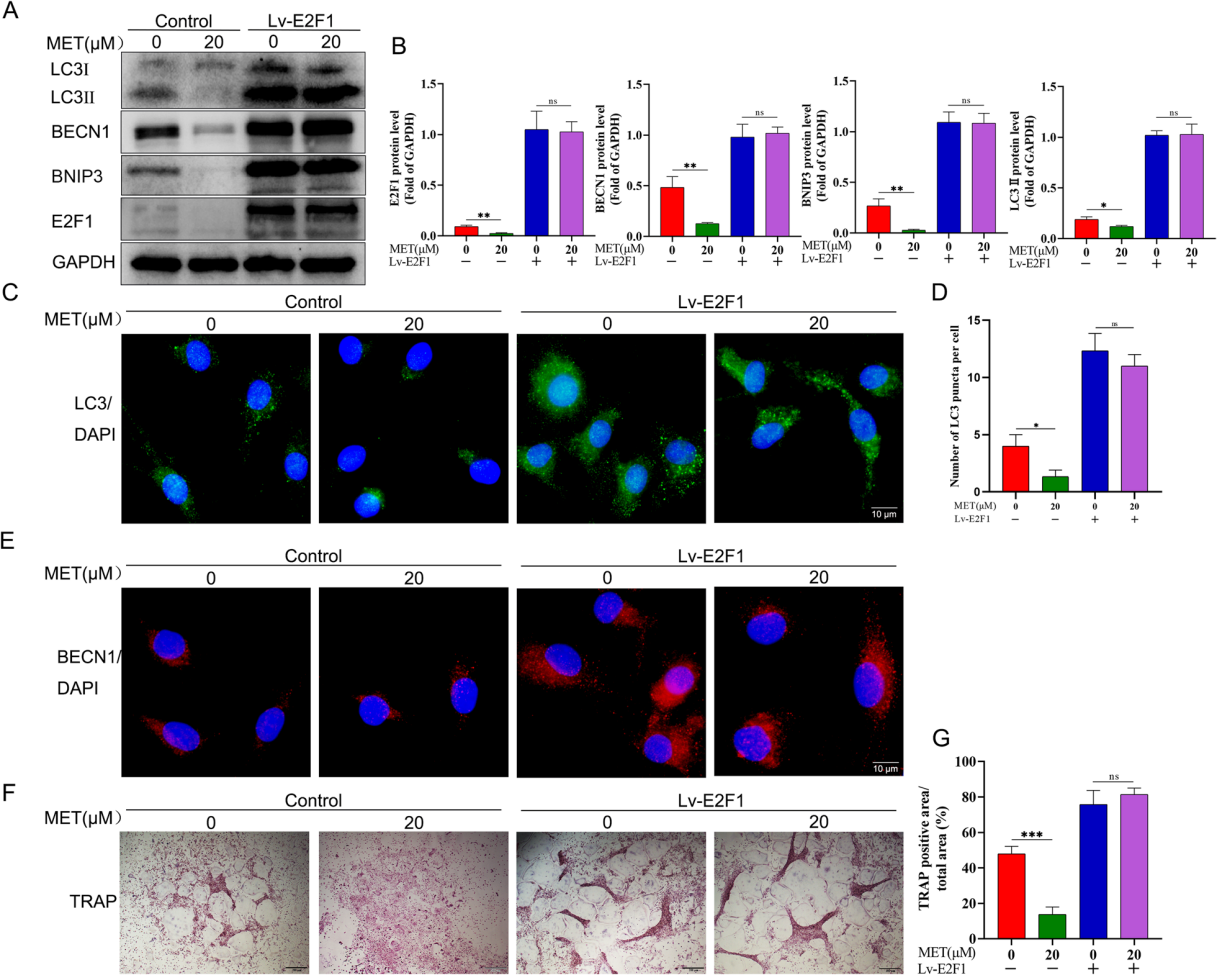
Metformin-mediated autophagy downregulation via BECN1 and BNIP3 is E2F1 dependent. **A** Cell lysates of osteoclast precursors were used to examine the expression of E2F1, BNIP3 and LC3 proteins by western bolt. After overexpression of E2F1 (Lv-E2F1), osteoclast precursors were treated with metformin (0, 20 μM) for 5 d in the presence of 30 ng/mL M-CSF and 50 ng/mL RANKL. **B** The quantitative analysis of immunoblots relative to GAPDH protein level in (**A**). **C** After overexpression of E2F1 (Lv-E2F1), osteoclast precursors were treated with metformin (0, 20 μM) for 24 h, and immunofluorescence staining for LC3 was performed. **D** Quantification of the LC3 puncta per cell in (**C**). **E** After overexpression of E2F1 (Lv-E2F1), osteoclast precursors were treated with metformin (0, 20 μM) for 24 h, immunofluorescent staining (IF) analyses of osteoclast precursors using anti-BECN1 antibody. (F, G) After osteoclast precursors were treated as in (**A**), TRAP staining was performed and TRAP-positive multinucleated osteoclasts (≥ 3 nuclei) were counted. Values displayed are means ± SD of 3 independent experiments (*p < 0.05; **p < 0.01; ***p < 0.001, ns, non-significant)

Although E2F1 is widely accepted as playing a crucial role in cell cycle and metabolism [37], a recent study revealed that the E2F1 level in postmenopausal women with high BMD is significantly decreased as compared with that in postmenopausal women with low BMD based on exon array analysis in peripheral blood monocytes (PBM) [45]. Therefore, it is very likely that E2F1 plays an important role in osteoclast formation and activity. We next examined the role of E2F1 in the regulation of autophagy by BECN1 and BNIP3. As shown in Fig. 7A, B, metformin treatment obviously decreased the expression level of E2F1 compared with RANKL treatment only, followed by reduced expression levels of BECN1, BNIP3, and LC3II. To further ascertain the role of E2F1 in metformin-downregulated autophagy during osteoclast differentiation, we tested whether overexpression of E2F1 could abolish the reduction in metformin-mediated autophagy. Interestingly, compared with control group, lentivirus-mediated E2F1 overexpression in osteoclast precursors in the presence of M-CSF and RANKL not only sharply upregulated BECN1, BNIP3 and LC3II expression levels, but also markedly enhanced the accumulation of LC3 puncta and BECN1fluorescence intensity, and finally reduced BECN1, BNIP3 and LC3-II expression levels mediated by metformin were abolished (Fig. 7A–E). Moreover, overexpression of E2F1 also resulted in significantly elevated osteoclast formulation as compared with the control group, while the effect could not be alleviated by metformin (Fig. 7F, G), which was consistent with LC3 puncta and BECN1fluorescence intensity in osteoclast precursors. On the whole, these results suggested that the downregulation of autophagy after metformin treatment in osteoclast precursors is mediated by the reduction of E2F1 levels, and E2F1 is required for the inhibition of the autophagy upon downregulation of BECN1 and BNIP3 levels.

### E2F1-siRNA administration rescues OVX-related bone loss in vivo

**Fig. 8:**
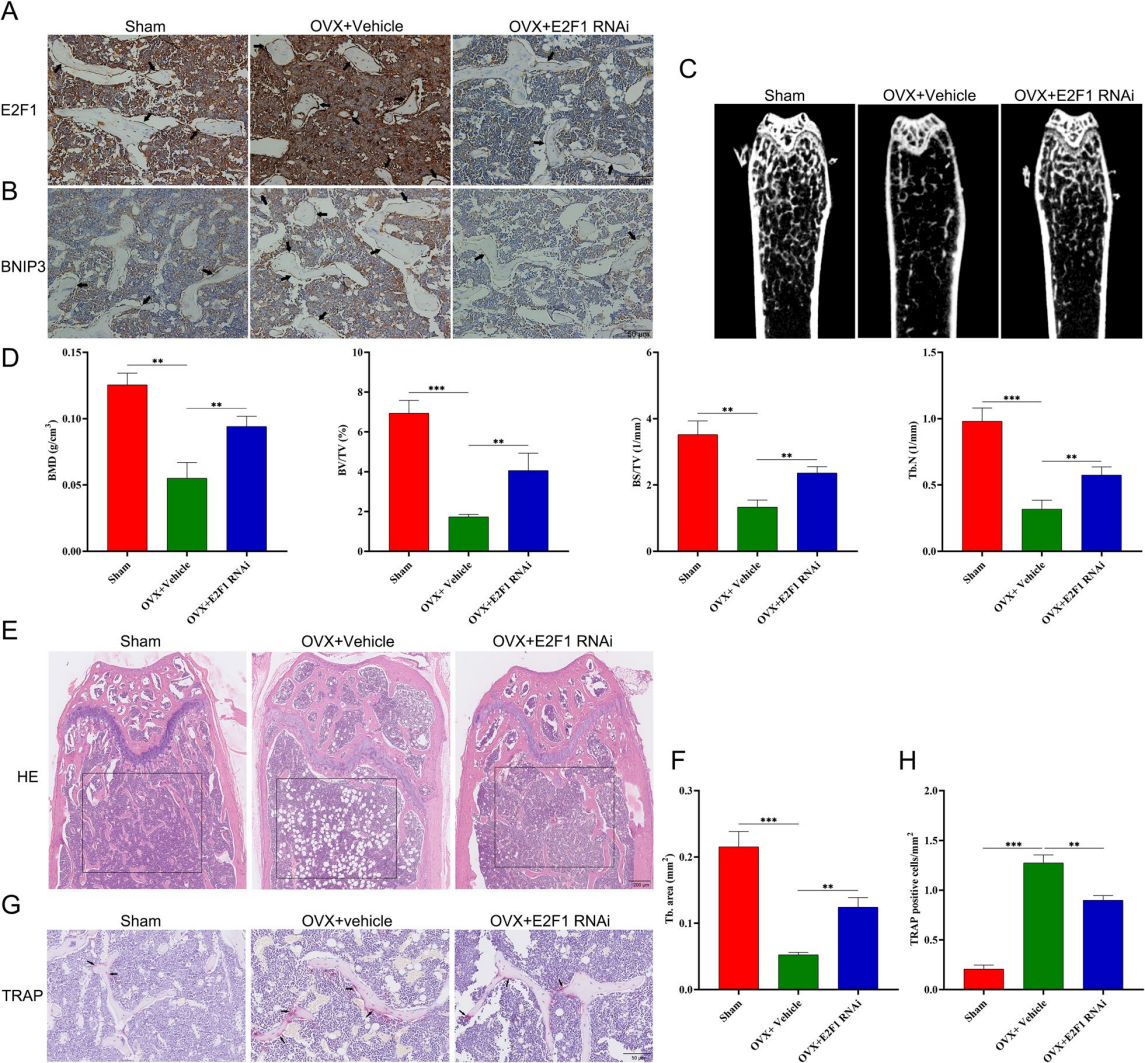
E2F1-siRNA administration rescues OVX-related bone loss in vivo. Mice were treated with Vehicle (Saline) or E2F1-RNAi (250 nmol/kg) through tail vein twice per week for 4 weeks after four weeks following ovariectomy or sham surgery. **A** Representative immunohistochemical staining for E2F1 in distal femur sections from sham (n = 3), OVX + vehicle (n = 3) and OVX + E2F1 RANi (E2F1 siRNA; n = 3). **B** Representative immunohistochemical staining for BNIP3 in distal femur sections from sham (n = 3), OVX + Vehicle (n = 3) and OVX + E2F1 RANi (n = 3). **C** Micro CT analysis of the distal femur from sham, OVX + Vehicle and OVX + E2F1 RNAi group. **D** Calculations of bone mineral density (BMD), bone value/total value (BV/TV), bone surface area/total value (BS/TV) and trabecular number (Tb.N). **E** Histological H&E staining of distal femur sections. (F) Quantitative histomorphometric assessment of trabecular number (Tb. N) based on H&E-stained femur sections. **G** Histological TRAP staining of femur sections. **H** Quantitative assessment of osteoclasts number based on TRAP-stained tibial sections. Values displayed are means ± SD of 3 independent experiments (*p < 0.05; **p < 0.01; ***p < 0.001)

In order to test the effects of E2F1 in vivo inhibition on OVX-induced bone loss, ovariectomized mice were treated with E2F1-siRNA for 4 weeks. To examine the effect of E2F1 administration, we performed immunohistochemical analysis for E2F1 and BNIP3 levels, and the results revealed that treatment with E2F1-siRNA dramatically knocked down the expression of E2F1, followed by decreasing BNIP3 level in femurs as compared with vehicle-treated ovariectomized mice (Fig. 8A, B), which demonstrated that E2F1-siRNA administration via tail-vein was effective method. Relative to the vehicle-treated group, E2F1 siRNA-treated ovariectomized mice had better trabecular bone microarchitecture in the femur (Fig. 8C). Morphometric analyses of trabecular parameters confirmed the decreased bone mass in BMD, BS/TV, BV/TV, and Tb.N in ovariectomized mice, while the OVX-induced deterioration of the trabecular microarchitecture could be reversed by E2F1 siRNA administration (Fig. 8D). Furthermore, the bone tissue microarchitecture was evaluated by histological staining of tissue sections with H&E. The results showed that the low-bone mass phenotype in ovariectomized mice was obvious in H&E-stained tissue samples (Fig. 8E) and this was verified by quantitative histomorphometric analysis of Tb.N (Fig. 7F). There is growing awareness that excessive osteoclast formation and bone resorption are often the latent causes of bone loss after the menopause. Therefore, we performed TRAP staining of femur bone sections to assess osteoclast numbers and activity. As shown in Fig. 8G and H, femur sections from ovariectomized mice administered E2F1 siRNA had presented decreased numbers of osteoclasts than other ovariectomized mice. Together, these results showed that silencing of E2F1 in ovariectomized mice resultes in amelioration of OVX-induced bone loss.

## Discussion

**Fig. 9:**
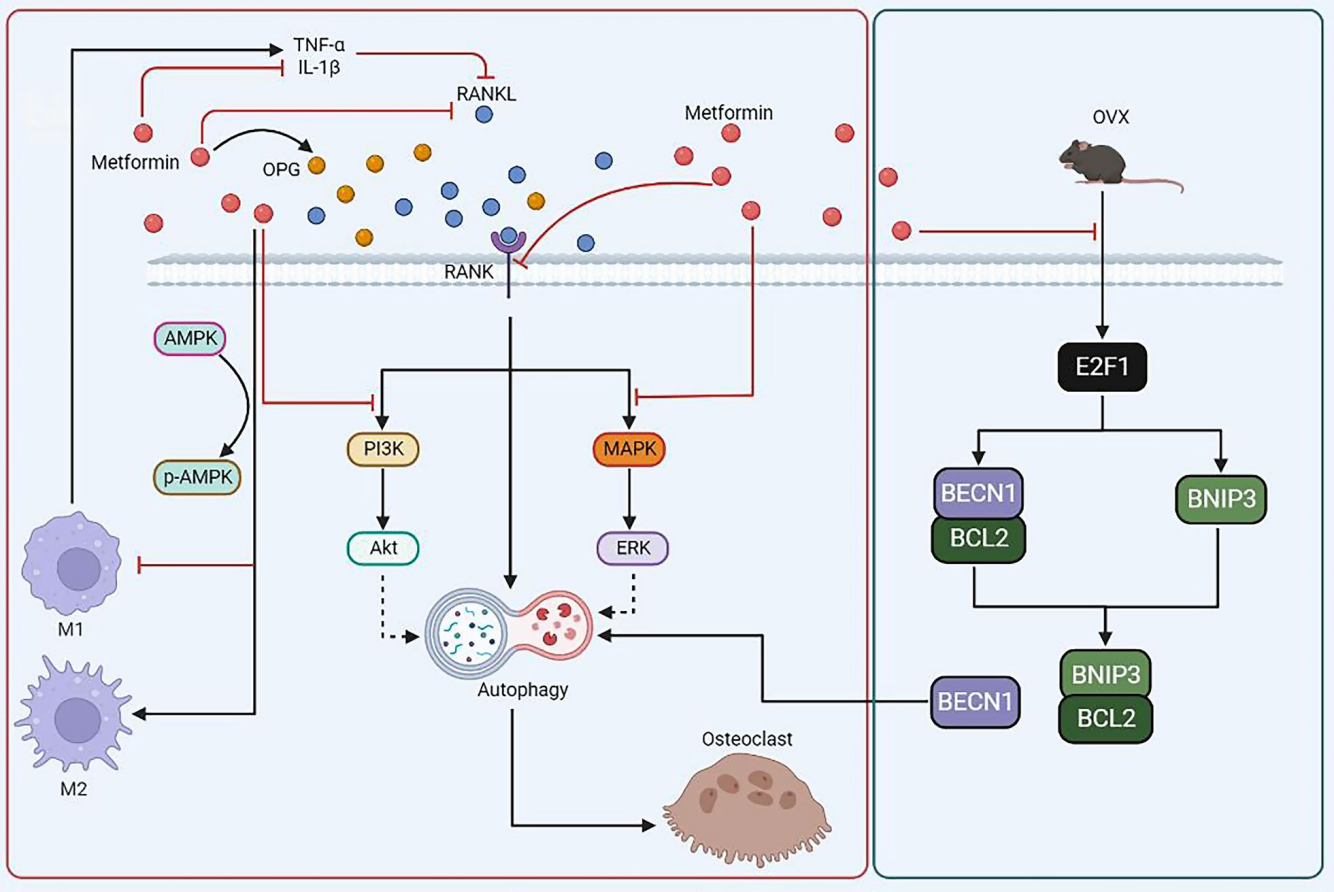
Schematic showing that the molecular mechanism underlying the inhibition of osteoclastogenesis by metformin in our study (Green box), and the possible relationship between autophagy and other signaling pathways involving in osteoclast differentiation and function upon metformin stimulation in previous studies (red box). **A** Metformin treatment inhibits OVX-induced E2F1 upregulation. Then, metformin downregulates OVX-induced BECN1 and BNIP3 overexpression, followed by reduction of the connection between BNIP3 and BCL2, which subsequently promotes the association of BECN1 and BCL2, resulting in reduction of the free BECN1 level. In this way, OVX-triggered autophagy upregulation is suppressed by metformin, leading to the alleviation of OVX-induced bone loss. **B** Increased level of autophagy was observed during the RANKL-induced osteoclastogenesis. (a, b, c) Metformin was demonstrated to downregulate the expression level of RANK (a), RANKL (b) and OPG (c). (d) Inflammation factors, such as TNF-α and IL-1β, could reduce RANKL expression. (e) Metformin was shown to downregulate the level of TNF-α and IL-1β. (f, g) Metformin could promote the polarization of anti-inflammatory M2 macrophages and inhibit the polarization of proinflammatory M1 macrophages (f), followed by reduction of TNF-α and IL-1β level (g). (h, i) Metformin could inhibit the PI3K/Akt (h) and MAPK/ERK (i) signaling pathways during osteoclastogenesis

In this study, we showed for the first time that metformin exerts a bone protective action against postmenopausal bone loss through inhibition of OVX-induced autophagosome synthesis during osteoclastogenesis. This process is mediated by the E2F1-BNIP3 and BECN1 signaling pathways, instead of a conventional metformin-AMPK pathway. Mechanistically, metformin treatment inhibited estrogen deficiency-induced E2F1 upregulation, followed by downregulated expression levels of BECN1 and BNIP3 protein, which subsequently suppressed the binding between BNIP3 and BCL2, thus promoting the association of BECN1 and BCL2. In this way, OVX-activated autophagy is downregulated by metformin, leading to the alleviation of OVX-induced bone loss (Fig. 9A).

It is well established that metformin has a protective effect on bone health [25]. With our initial findings, we also confirmed that metformin use reduced bone resorption and improved bone quality in postmenopausal women and ovariectomized mice. Furthermore, we also observed that the beneficial effect on bone is achieved at least in part via decreased osteoclast formation and activity. In addition, the protective effect of metformin may be also attributed to activation of osteogenic differentiation via RUNX2 and AMP-activated protein kinase (AMPK)/upstream transcription factor 1(USF1)/small heterodimer partner (SHIP) signaling [23–25], activation of AMPK signaling pathway and subsequent enchancement in eNOS and BMP-2 production33 in MC3T3-E1 cells [46], inbibition of GSK3β in human mesenchymal stem cells (hBMSCs) [47] and decrease in reactive oxygen species level and adipogenic differentiation in adipose-drived multipotent mesenchymal stem cells in vitro [48]. Moreover, metformin treatment activates peroxisome proliferator–activated receptor γ coactivator 1-α (PGC-1α) in osteoblasts, promoting osteoblast survival and function in high-glucose condition and reversing osteopenia in diabetic mice [49].

We found that other mechanisms than OPG/RANKL may be involved in this process when we investigated the change in differentiation capacity in osteoclast precursors after metformin treatment in the presence of 30 ng/ml M-CSF and 50 ng/ml RANKL in vitro. Then, given that induction of autophagy is crucial for osteoclastic differentiation of monocytes [50], autophagic flux in osteoclast precursors from femurs and tibias of ovariectomized mice with or without metformin treatment or osteoclast precursors after metformin treatment were detected, and we found that the increased autophagy level is associated with  increased osteoclast formation and bone resorption in postmenopausal bone loss and metformin treatment sharply downregulated the autophagic level. These findings were consistent with previous reports that specific deletion of Atg7 in monocytes and systemic delivery of chloroquine, an autophagy inhibitor, could decrease osteoclast activity and finally alleviate bone loss in ovariectomized mice [14]. However, the role of autophagy in osteoclast and osteoblast activity after metformin stimulation is uncertain. Autophagy upregulation is related to the osteogenic differentiation of MSCs [45], and estrogen deficiency downregulates autophagic activity in osteoblasts, promoting the development of osteoporosis [51]. These studies showed that activation of autophagy in osteoblast precursors is beneficial in mitigating estrogen deficiency-induced osteoporosis. However, whether metformin promotes/inhibits autophagy in osteoblasts was not explored further in this study. Nevertheless, it is reasonable to believe that the downregulated autophagy level in osteoclast precursors mediated by metformin plays a dominant role in reversing the postmenopausal bone loss in this study. In addition, previous studies also showed that metformin treatment could inhibit osteoclast formation and activity through PI3K/Akt and ERK signaling [31], decreasing the inflammatory response and oxidative stress [52], reducing the RANK expression in osteoclast precursors [53], promoting the polarization of macrophages to an anti-inflammatory phenotype [54], reducing RANKL expression and stimulating OPG expression in osteoblasts [17, 26], and prevent bone loss. Furthermore, previous studies indicated that PI3K/Akt and ERK signaling [55–57], inflammation factors signaling [58], OPG/RANKL signalingand RANK signaling [56, 59] might be also involving in regulating autophagic level during osteoclast differentiation and bone resorption (Fig. 9B). Therefore, autophagy might play a crucial role in the inhibition of bone loss by metformin.

We next investigated the involvement of metformin in autophagy more specifically to identify the regulatory target in osteoclast precursors. E2F1 was first identified as a protein that belongs to the E2F family, which is known for binding to the retinoblastoma protein (pRB), a tumor inhibitor mutated in many types of cancer [60, 61]. Recently, it was also reported that E2F1 was involved in regulation of osteoclastogensis and autophagy [62–65]. For example, E2F1 promoted osteoclastogensis by regulating glycolytic metabolism [62]. In searching for factors associated with low BMD in postmenopausal women, Zhou et al. reported that downregulated E2F1 was related to higher BMD in the hip [45]. In addition, it is known that E2F1 is involved in regulating the autophagy process in some tumors [62, 63, 65], but whether it participates in regulation of autophagy activity in osteoclast precursors remains unclear. Indeed, it was found that the level of E2F1 was decreaced in response to metformin stimulation, while overexpression of E2F1 abolished the suppressive effect of metformin on RANKL-induced autophagy level in osteoclast precursors, followed by restoring metformin-associated inhibition of osteoclast differentiation. Furthermore, E2F1 silencing effectively suppressed osteoclastogensis in vivo.

BECN1, a novel Bcl-2-homology (BH)-3 domain-only protein, is considered as one of the most important regulators of autophagy in the autophagy process [66, 67]. However, BECN1-induced autophagy would be inhibited when BECN1 is bound to BCL2 [68]. In this study, we have focused on BECN1 to specifically determine the regulation mechanism of metformin in the autophagy process. Indeed, we observed that metformin-mediated E2F1 downregulation not only suppressed BECN1 expression but also reduced the expression of the BNIP3 protein that competitively binds to BECN1 with BCL2 through binding with BECN1 and BNIP3 promotors [66, 69]. Furthermore, dissociation of BNCN1 from BECN1/BCL2 complex was reduced, and autophagy was downregulated, leading to inhibition of osteoclastogenesis.

However, there are some limitations in our present study. Among these, one of the most important is that there might be some side effects associated with metformin or E2F1 siRNA therapy when the systemic use of metformin or E2F1 siRNA. Based on this, the problem could be addressed through at least three methods as follows: (1) Metformin or E2F1 siRNA was loaded in biomaterials targeted bone or osteoclasts [70, 71], such as CXCR4 + exosomes [72] and alendronate functionalized nanoparticles [73]. (2) Local administration of metformin or E2F1 siRNA in some osteoporotic disorders, such as intertrochanteric fractures and vertebral fractures. (3) Bone-target modification for metformin or E2F1 siRNA [74, 75]. Together, the above methods may be better therapeutic choices than modes of traditional administration, which seem to reduce the off-target effects.

## Conclusion

Collectively, our study confirmed that the beneficial effects of metformin on postmenopausal bone loss could be attributed, at least in part, to its role in downregulating the OVX-activated autophagy level during osteoclast formation. Mechanistically, we also demonstrated that the inhibition of  OVX-activated autophagy level upon metformin treatment was associated with  E2F1-BECN1 and E2F1-BNIP3 signaling pathway. These insights about metformin will provide clues for future treatment of postmenopausal osteoporosis through modulating E2F1.

## Supplementary Information

**Additional file 1.** Supplementary material.

## Data Availability

The data and material for this study are available on request form the corresponding author.

## Abbreviations

β-CTX: C-terminal telopeptide of type I collagen β isomer
TRAP-5b: Tartrate-resistant acid phosphatase type 5b
TRAP: Tartrate-resistant acid phosphatase
BMD: Bone mineral density
BV/TV: Bone volume per tissue volume
Tb.N: Trabecular number
LC3: Microtubule-associated protein 1 light chain 3
Beclin1: Autophagy related
CTSK: Cathepsin K
NFATc1: Nuclear factor of activated T cells 1
ACP5: Acid phosphatase 5
Micro-CT: Microcomputed tomography
qRT-PCR: Quantitative reverse-transcriptase polymerase chain reaction
Co-IP: Co-immunoprecipitation
GFP: Enhanced green fluorescent protein
M-CSF: Macrophage colony-stimulating factor
RANKL: Receptor activator of nuclear factor-κB (RANK) ligand
PM: Postmenopausal women
MET: Metformin
T2DM: Type 2 diabetes mellitus

## References

[1] Boyle WJ, Simonet WS, Lacey DL (2003). Osteoclast differentiation and activation. Nature.

[2] Compston JE, McClung MR, Leslie WD (2019). Osteoporosis. Lancet.

[3] Cosman F, de Beur SJ, LeBoff MS, Lewiecki EM, Tanner B, Randall S (2014). Clinician's guide to prevention and treatment of osteoporosis. Osteoporos Int.

[4] Tella SH, Gallagher JC (2014). Prevention and treatment of postmenopausal osteoporosis. J Steroid Biochem Mol Biol.

[5] Reid IR (2015). Short-term and long-term effects of osteoporosis therapies. Nat Rev Endocrinol.

[6] Teitelbaum SL, Ross FP (2003). Genetic regulation of osteoclast development and function. Nat Rev Genet.

[7] Lagasse E, Weissman IL (1997). Enforced expression of Bcl-2 in monocytes rescues macrophages and partially reverses osteopetrosis in op/op mice. Cell.

[8] Kong YY, Yoshida H, Sarosi I, Tan HL, Timms E, Capparelli C (1999). OPGL is a key regulator of osteoclastogenesis, lymphocyte development and lymph-node organogenesis. Nature.

[9] Levine B, Kroemer G (2008). Autophagy in the pathogenesis of disease. Cell.

[10] Mizushima N, Levine B, Cuervo AM, Klionsky DJ (2008). Autophagy fights disease through cellular self-digestion. Nature.

[11] DeSelm CJ, Miller BC, Zou W, Beatty WL, van Meel E, Takahata Y (2011). Autophagy proteins regulate the secretory component of osteoclastic bone resorption. Dev Cell.

[12] Wang K, Niu J, Kim H, Kolattukudy PE (2011). Osteoclast precursor differentiation by MCPIP via oxidative stress, endoplasmic reticulum stress, and autophagy. J Mol Cell Biol.

[13] Shi J, Wang L, Zhang H, Jie Q, Li X, Shi Q (2015). Glucocorticoids: Dose-related effects on osteoclast formation and function via reactive oxygen species and autophagy. Bone.

[14] Lin NY, Chen CW, Kagwiria R, Liang R, Beyer C, Distler A (2016). Inactivation of autophagy ameliorates glucocorticoid-induced and ovariectomy-induced bone loss. Ann Rheum Dis.

[15] Hidayat K, Du X, Wu MJ, Shi BM (2019). The use of metformin, insulin, sulphonylureas, and thiazolidinediones and the risk of fracture: Systematic review and meta-analysis of observational studies. Obes Rev.

[16] Blümel JE, Arteaga E, Aedo S, Arriola-Montenegro J, López M, Martino M (2020). Metformin use is associated with a lower risk of osteoporosis in adult women independent of type 2 diabetes mellitus and obesity. REDLINC IX study. Gynecol Endocrinol.

[17] Mai QG, Zhang ZM, Xu S, Lu M, Zhou RP, Zhao L (2011). Metformin stimulates osteoprotegerin and reduces RANKL expression in osteoblasts and ovariectomized rats. J Cell Biochem.

[18] Son HJ, Lee J, Lee SY, Kim EK, Park MJ, Kim KW (2014). Metformin attenuates experimental autoimmune arthritis through reciprocal regulation of Th17/Treg balance and osteoclastogenesis. Mediators Inflamm.

[19] Matsuoka Y, Morimoto S, Fujishiro M, Hayakawa K, Kataoka Y, Suzuki S (2021). Metformin repositioning in rheumatoid arthritis. Clin Exp Rheumatol.

[20] Xie Z, Lau K, Eby B, Lozano P, He C, Pennington B (2011). Improvement of cardiac functions by chronic metformin treatment is associated with enhanced cardiac autophagy in diabetic OVE26 mice. Diabetes.

[21] Li J, Gui Y, Ren J, Liu X, Feng Y, Zeng Z (2016). Metformin protects against cisplatin-induced tubular cell apoptosis and acute kidney injury via AMPKα-regulated autophagy induction. Sci Rep.

[22] Son SM, Shin HJ, Byun J, Kook SY, Moon M, Chang YJ (2016). Metformin facilitates amyloid-β generation by β- and γ-secretases via autophagy activation. J Alzheimers Dis.

[23] Shah M, Kola B, Bataveljic A, Arnett TR, Viollet B, Saxon L (2010). AMP-activated protein kinase (AMPK) activation regulates in vitro bone formation and bone mass. Bone.

[24] Sofer E, Shargorodsky M (2016). Effect of metformin treatment on circulating osteoprotegerin in patients with nonalcoholic fatty liver disease. Hepatol Int.

[25] Bahrambeigi S, Yousefi B, Rahimi M, Shafiei-Irannejad V (2019). Metformin; an old antidiabetic drug with new potentials in bone disorders. Biomed Pharmacother.

[26] Liu L, Zhang C, Hu Y, Peng B (2012). Protective effect of metformin on periapical lesions in rats by decreasing the ratio of receptor activator of nuclear factor kappa B ligand/osteoprotegerin. J Endod.

[27] Guo H, Ding D, Wang L, Yan J, Ma L, Jin Q (2021). Metformin attenuates osteoclast-mediated abnormal subchondral bone remodeling and alleviates osteoarthritis via AMPK/NF-κB/ERK signaling pathway. PLoS ONE.

[28] Xie H, Cui Z, Wang L, Xia Z, Hu Y, Xian L (2014). PDGF-BB secreted by preosteoclasts induces angiogenesis during coupling with osteogenesis. Nat Med.

[29] Chen X, Zhi X, Pan P, Cui J, Cao L, Weng W (2017). Matrine prevents bone loss in ovariectomized mice by inhibiting RANKL-induced osteoclastogenesis. FASEB J.

[30] Miyauchi Y, Sato Y, Kobayashi T, Yoshida S, Mori T, Kanagawa H (2013). HIF1α is required for osteoclast activation by estrogen deficiency in postmenopausal osteoporosis. Proc Natl Acad Sci USA.

[31] Bian F, Zhang Y, Xie Y, Fang H, Fan M, Wang X (2021). Effects of different concentrations of metformin on osteoclast differentiation and apoptosis and its mechanism. Pharmazie.

[32] Pierrefite-Carle V, Santucci-Darmanin S, Breuil V, Camuzard O, Carle GF (2015). Autophagy in bone: Self-eating to stay in balance. Ageing Res Rev.

[33] Shapiro IM, Layfield R, Lotz M, Settembre C, Whitehouse C (2014). Boning up on autophagy: the role of autophagy in skeletal biology. Autophagy.

[34] Guo J, Ren R, Sun K, Yao X, Lin J, Wang G (2020). PERK controls bone homeostasis through the regulation of osteoclast differentiation and function. Cell Death Dis.

[35] Foretz M, Guigas B, Bertrand L, Pollak M, Viollet B (2014). Metformin: from mechanisms of action to therapies. Cell Metab.

[36] Wang Y, Xu W, Yan Z, Zhao W, Mi J, Li J (2018). Metformin induces autophagy and G0/G1 phase cell cycle arrest in myeloma by targeting the AMPK/mTORC1 and mTORC2 pathways. J Exp Clin Cancer Res.

[37] Liang XH, Jackson S, Seaman M, Brown K, Kempkes B, Hibshoosh H (1999). Induction of autophagy and inhibition of tumorigenesis by beclin 1. Nature.

[38] Arai A, Kim S, Goldshteyn V, Kim T, Park NH, Wang CY (2019). Beclin1 modulates bone homeostasis by regulating osteoclast and chondrocyte differentiation. J Bone Miner Res.

[39] Feng Y, He D, Yao Z, Klionsky DJ (2014). The machinery of macroautophagy. Cell Res.

[40] Jung YY, Lee YK, Koo JS (2016). The potential of Beclin 1 as a therapeutic target for the treatment of breast cancer. Expert Opin Ther Targets.

[41] Meijer WH, van der Klei IJ, Veenhuis M, Kiel JA (2007). ATG genes involved in non-selective autophagy are conserved from yeast to man, but the selective Cvt and pexophagy pathways also require organism-specific genes. Autophagy.

[42] Kuma A, Komatsu M, Mizushima N (2017). Autophagy-monitoring and autophagy-deficient mice. Autophagy.

[43] Lei K, Davis RJ (2003). JNK phosphorylation of Bim-related members of the Bcl2 family induces Bax-dependent apoptosis. Proc Natl Acad Sci USA.

[44] Ma X, Godar RJ, Liu H, Diwan A (2012). Enhancing lysosome biogenesis attenuates BNIP3-induced cardiomyocyte death. Autophagy.

[45] Zhou Y, Zhu W, Zhang L, Zeng Y, Xu C, Tian Q (2018). Transcriptomic data identified key transcription factors for osteoporosis in Caucasian women. Calcif Tissue Int.

[46] Kanazawa I, Yamaguchi T, Yano S, Yamauchi M, Sugimoto T (2008). Metformin enhances the differentiation and mineralization of osteoblastic MC3T3-E1 cells via AMP kinase activation as well as eNOS and BMP-2 expression. Biochem Biophys Res Commun.

[47] Ma J, Zhang ZL, Hu XT, Wang XT, Chen AM (2018). Metformin promotes differentiation of human bone marrow derived mesenchymal stem cells into osteoblast via GSK3β inhibition. Eur Rev Med Pharmacol Sci.

[48] Marycz K, Tomaszewski KA, Kornicka K, Henry BM, Wroński S, Tarasiuk J (2016). Metformin decreases reactive oxygen species, enhances osteogenic properties of adipose-derived multipotent mesenchymal stem cells in vitro, and increases bone density in vivo. Oxid Med Cell Longev.

[49] Khan MP, Singh AK, Joharapurkar AA, Yadav M, Shree S, Kumar H (2015). Pathophysiological mechanism of bone loss in type 2 diabetes involves inverse regulation of osteoblast function by PGC-1α and skeletal muscle atrogenes: AdipoR1 as a potential target for reversing diabetes-induced osteopenia. Diabetes.

[50] Zhang Y, Morgan MJ, Chen K, Choksi S, Liu ZG (2012). Induction of autophagy is essential for monocyte-macrophage differentiation. Blood.

[51] Pernicova I, Kelly S, Ajodha S, Sahdev A, Bestwick JP, Gabrovska P (2020). Metformin to reduce metabolic complications and inflammation in patients on systemic glucocorticoid therapy: a randomised, double-blind, placebo-controlled, proof-of-concept, phase 2 trial. Lancet Diabetes Endocrinol.

[52] Araújo AA, Pereira A, Medeiros C, Brito GAC, Leitão RFC, Araújo LS (2017). Effects of metformin on inflammation, oxidative stress, and bone loss in a rat model of periodontitis. PLoS ONE.

[53] Tao LY, Łagosz-Ćwik KB, Hogervorst JMA, Schoenmaker T, Grabiec AM, Forouzanfar T (2021). Diabetes medication metformin inhibits osteoclast formation and activity in in vitro models for periodontitis. Front Cell Dev Biol.

[54] Yan Z, Tian X, Zhu J, Lu Z, Yu L, Zhang D (2018). Metformin suppresses UHMWPE particle-induced osteolysis in the mouse calvaria by promoting polarization of macrophages to an anti-inflammatory phenotype. Mol Med.

[55] Fu L, Wu W, Sun X, Zhang P (2020). Glucocorticoids enhanced osteoclast autophagy through the PI3K/Akt/mTOR signaling pathway. Calcif Tissue Int.

[56] Zhao H, Sun Z, Ma Y, Song R, Yuan Y, Bian J (2020). Antiosteoclastic bone resorption activity of osteoprotegerin via enhanced AKT/mTOR/ULK1-mediated autophagic pathway. J Cell Physiol.

[57] Xu X, Wang R, Wu R, Yan W, Shi T, Jiang Q (2020). Trehalose reduces bone loss in experimental biliary cirrhosis rats via ERK phosphorylation regulation by enhancing autophagosome formation. FASEB J.

[58] Liu W, Zhou J, Niu F, Pu F, Wang Z, Huang M (2020). Mycobacterium tuberculosis infection increases the number of osteoclasts and inhibits osteoclast apoptosis by regulating TNF-α-mediated osteoclast autophagy. Exp Ther Med.

[59] Xiu Y, Xu H, Zhao C, Li J, Morita Y, Yao Z (2014). Chloroquine reduces osteoclastogenesis in murine osteoporosis by preventing TRAF3 degradation. J Clin Invest.

[60] Bagchi S, Weinmann R, Raychaudhuri P (1991). The retinoblastoma protein copurifies with E2F-I, an E1A-regulated inhibitor of the transcription factor E2F. Cell.

[61] Dyson NJ (2016). RB1: a prototype tumor suppressor and an enigma. Genes Dev.

[62] Murata K, Fang C, Terao C, Giannopoulou EG, Lee YJ, Lee MJ (2017). Hypoxia-sensitive COMMD1 integrates signaling and cellular metabolism in human macrophages and suppresses osteoclastogenesis. Immunity.

[63] Ruan C, Wang C, Gong X, Zhang Y, Deng W, Zhou J (2021). An integrative multi-omics approach uncovers the regulatory role of CDK7 and CDK4 in autophagy activation induced by silica nanoparticles. Autophagy.

[64] Polager S, Ofir M, Ginsberg D (2008). E2F1 regulates autophagy and the transcription of autophagy genes. Oncogene.

[65] Jiang H, Martin V, Gomez-Manzano C, Johnson DG, Alonso M, White E (2010). The RB-E2F1 pathway regulates autophagy. Cancer Res.

[66] Kang R, Zeh HJ, Lotze MT, Tang D (2011). The Beclin 1 network regulates autophagy and apoptosis. Cell Death Differ.

[67] Oberstein A, Jeffrey PD, Shi Y (2007). Crystal structure of the Bcl-XL-Beclin 1 peptide complex: Beclin 1 is a novel BH3-only protein. J Biol Chem.

[68] Pattingre S, Tassa A, Qu X, Garuti R, Liang XH, Mizushima N (2005). Bcl-2 antiapoptotic proteins inhibit Beclin 1-dependent autophagy. Cell.

[69] Niu C, Chen Z, Kim KT, Sun J, Xue M, Chen G (2019). Metformin alleviates hyperglycemia-induced endothelial impairment by downregulating autophagy via the Hedgehog pathway. Autophagy.

[70] Zhou X, Cornel EJ, Fan Z, He S, Du J (2021). Bone-targeting polymer vesicles for effective therapy of osteoporosis. Nano Lett.

[71] Dou C, Li J, He J, Luo F, Yu T, Dai Q (2021). Bone-targeted pH-responsive cerium nanoparticles for anabolic therapy in osteoporosis. Bioact Mater.

[72] Hu Y, Li X, Zhang Q, Gu Z, Luo Y, Guo J (2021). Exosome-guided bone targeted delivery of Antagomir-188 as an anabolic therapy for bone loss. Bioact Mater.

[73] Chen Q, Zheng C, Li Y, Bian S, Pan H, Zhao X (2018). Bone targeted delivery of SDF-1 via alendronate functionalized nanoparticles in guiding stem cell migration. ACS Appl Mater Interfaces.

[74] Rotman SG, Grijpma DW, Richards RG, Moriarty TF, Eglin D, Guillaume O (2018). Drug delivery systems functionalized with bone mineral seeking agents for bone targeted therapeutics. J Control Release.

[75] Neale JR, Richter NB, Merten KE, Taylor KG, Singh S, Waite LC (2009). Bone selective effect of an estradiol conjugate with a novel tetracycline-derived bone-targeting agent. Bioorg Med Chem Lett.

