# Supplementary material for: Metformin alleviates bone loss in ovariectomized mice through inhibition of autophagy of osteoclast precursors mediated by E2F1
Source: Cell Commun Signal. 2022 Oct 25;20:165. doi: 10.1186/s12964-022-00966-5 (PMC9594975; doi:10.1186/s12964-022-00966-5)

**Supplementary material**

**Figure 6A:**

GAPDH


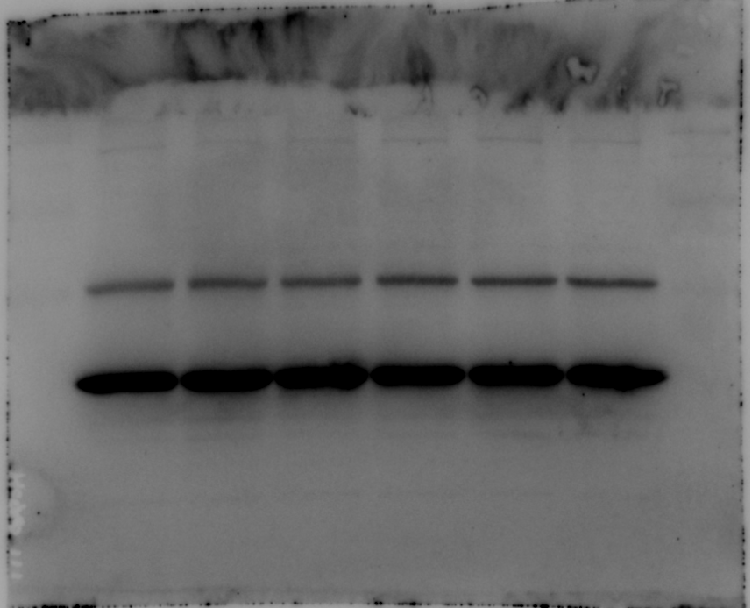

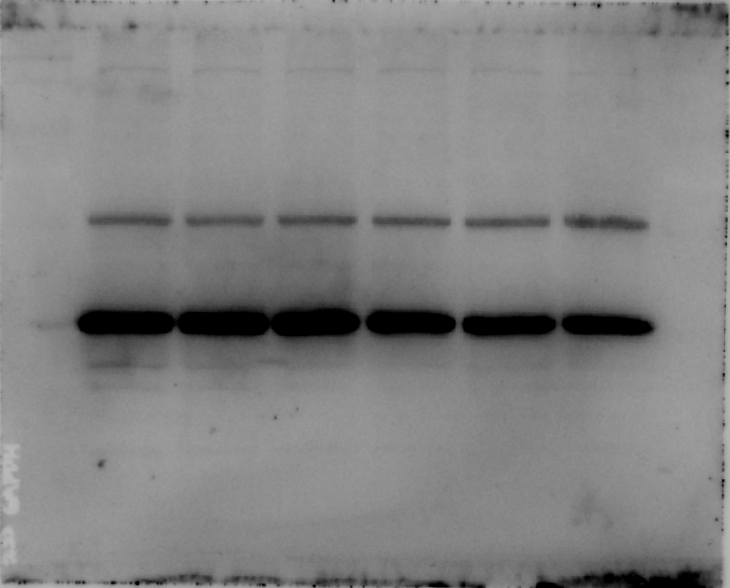

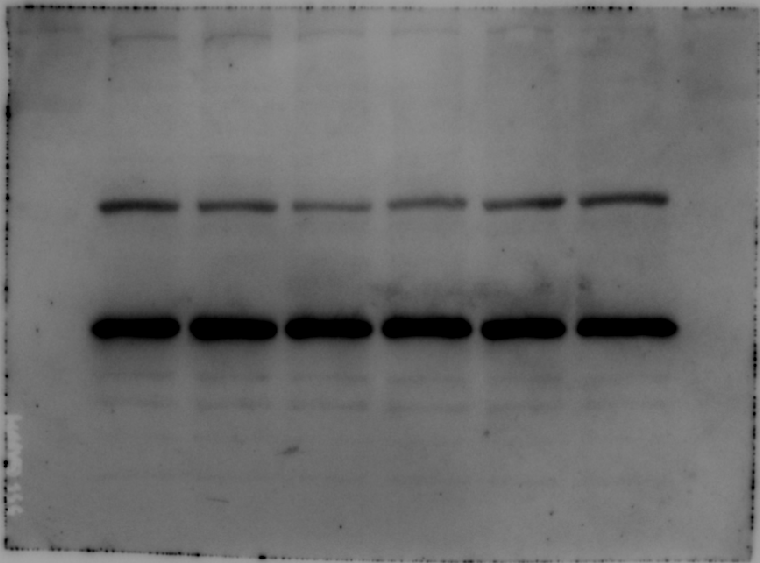


BECN1:


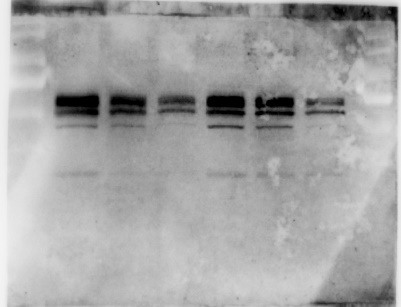

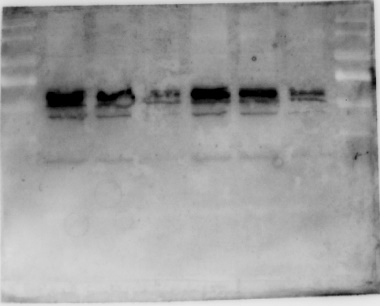

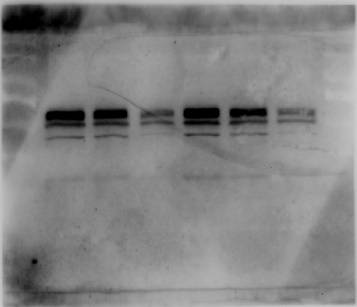


BNIP3


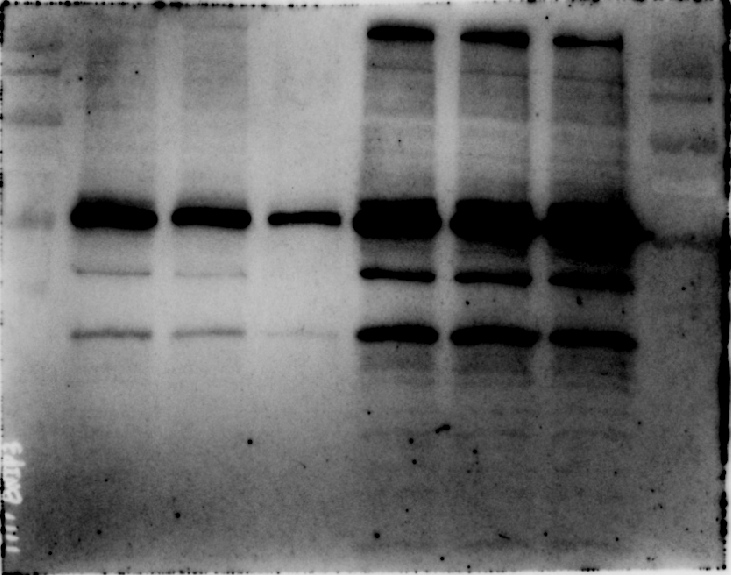

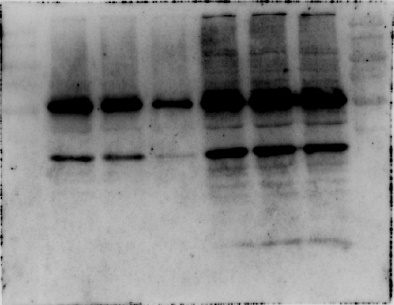

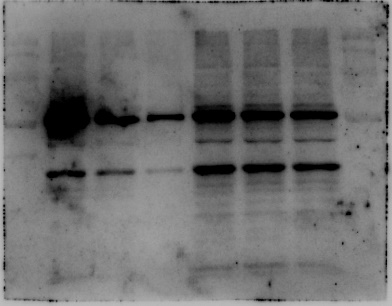


LC3


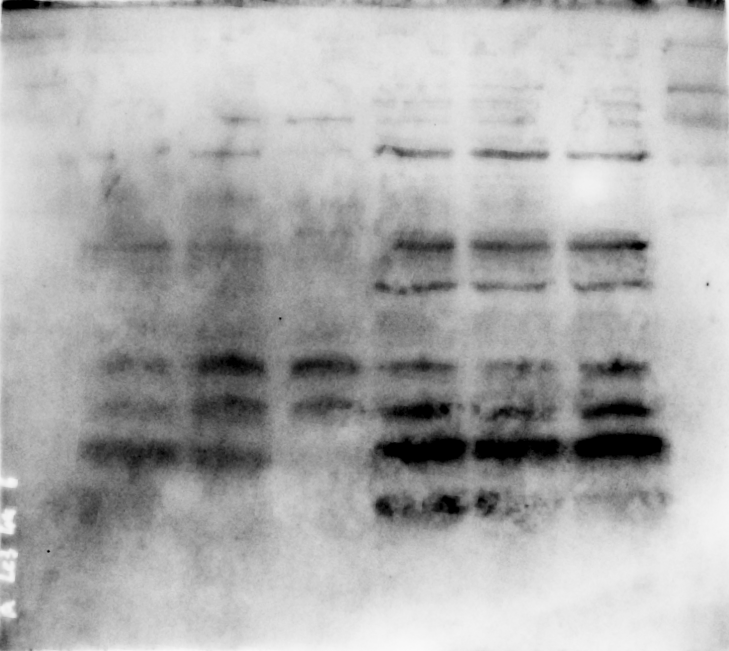

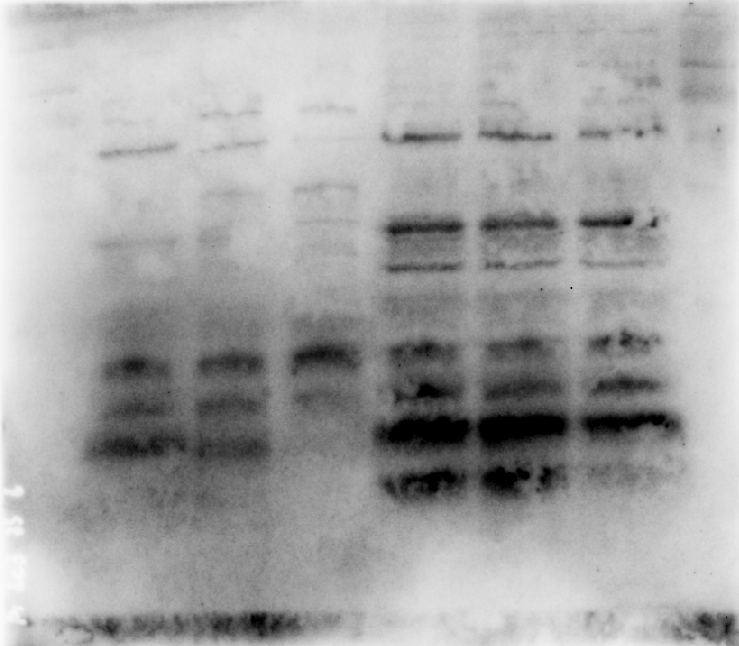

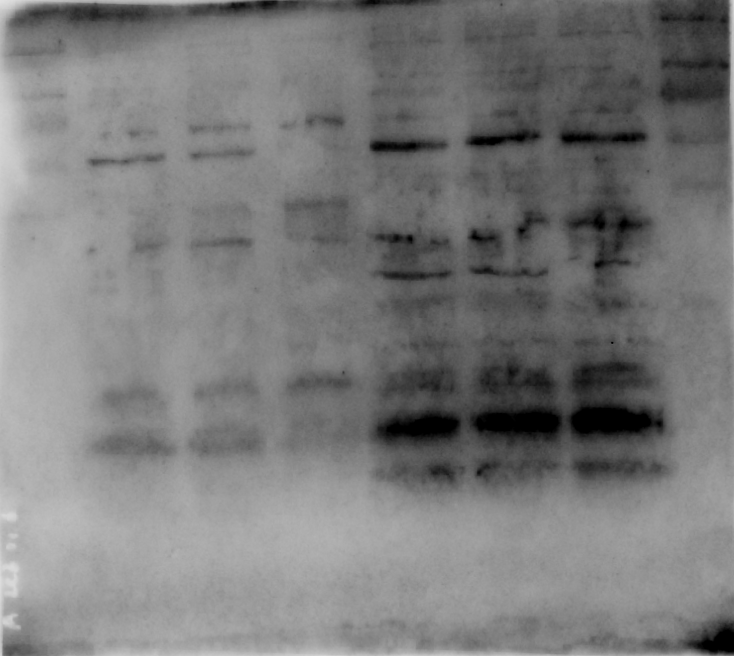


**Figure 6C**：

Input GAPDH


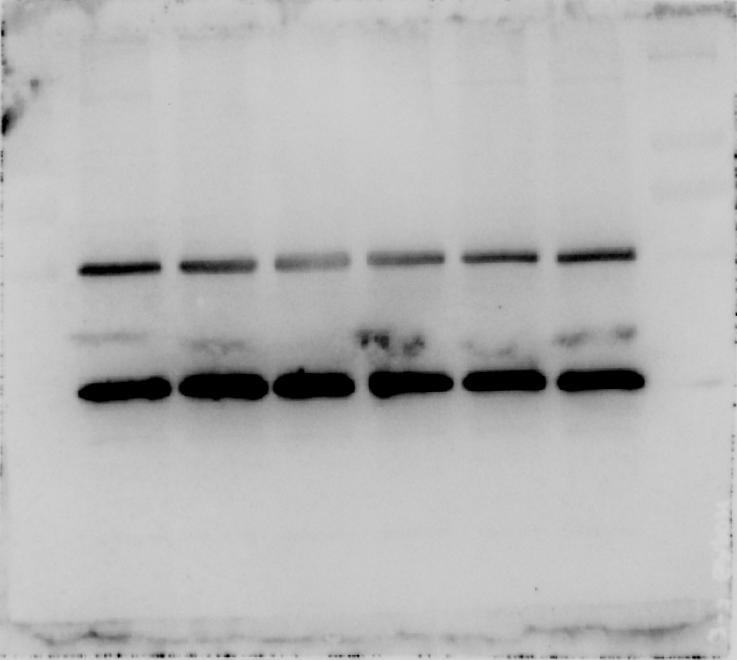

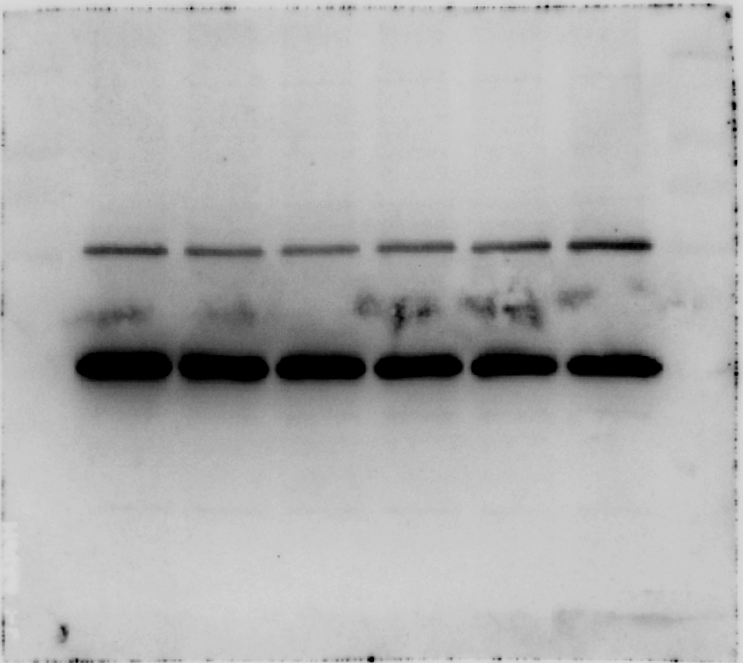

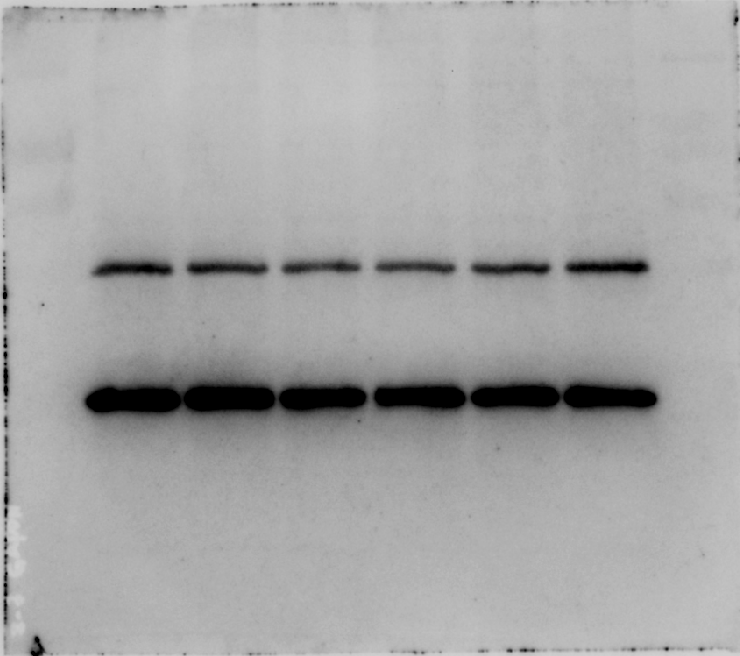


Input BNIP3


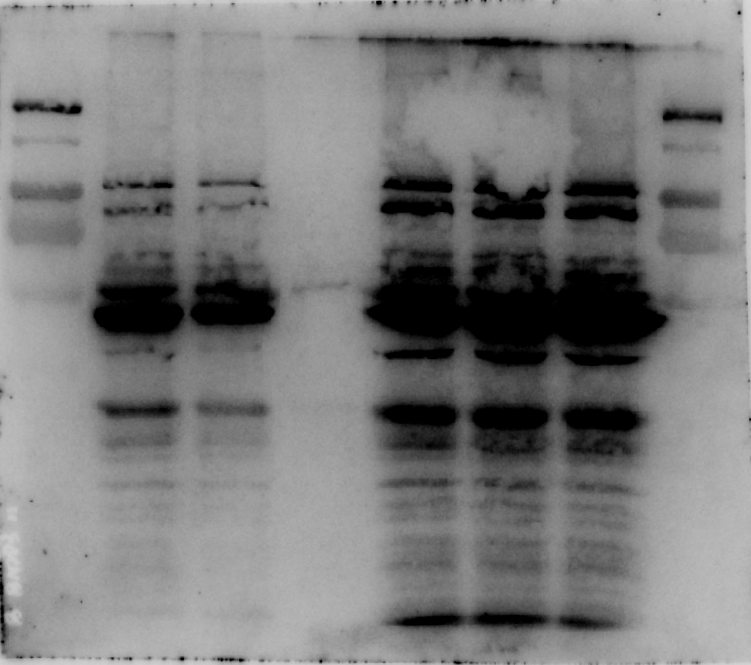

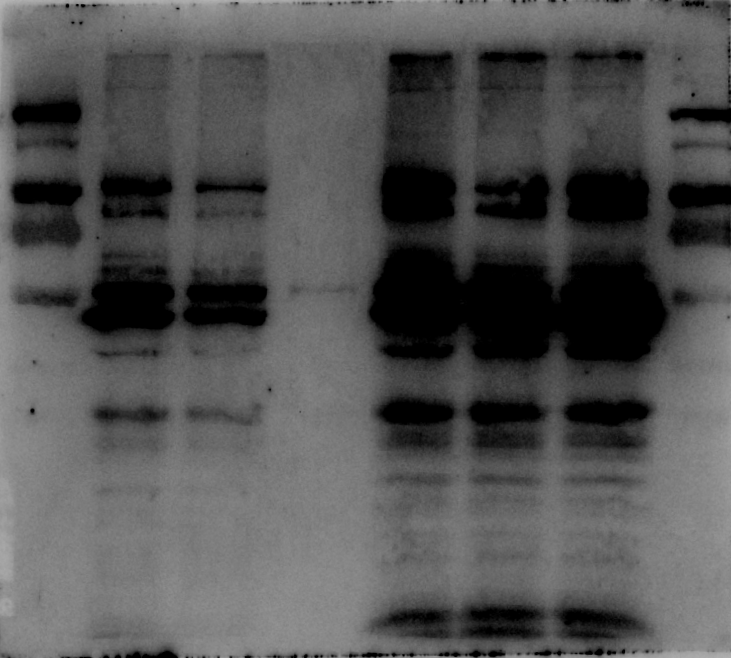

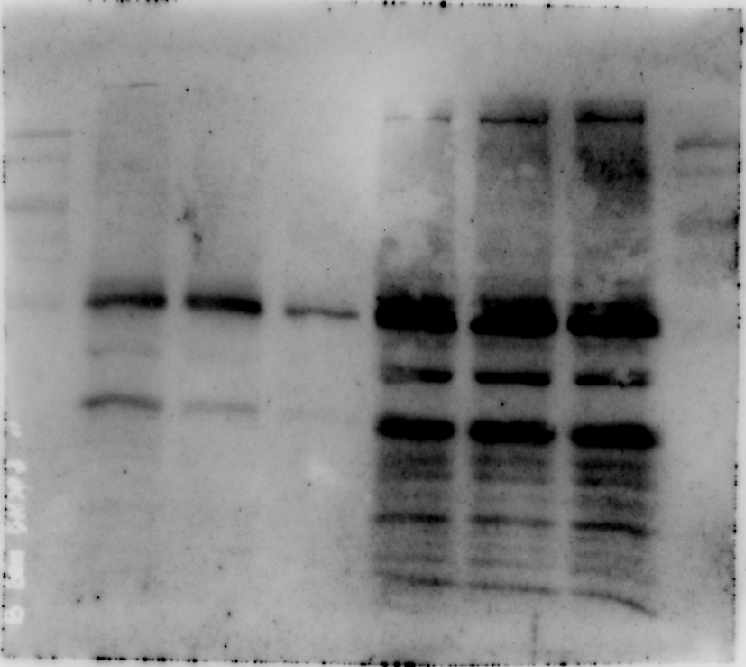


IP IgG：BNIP3


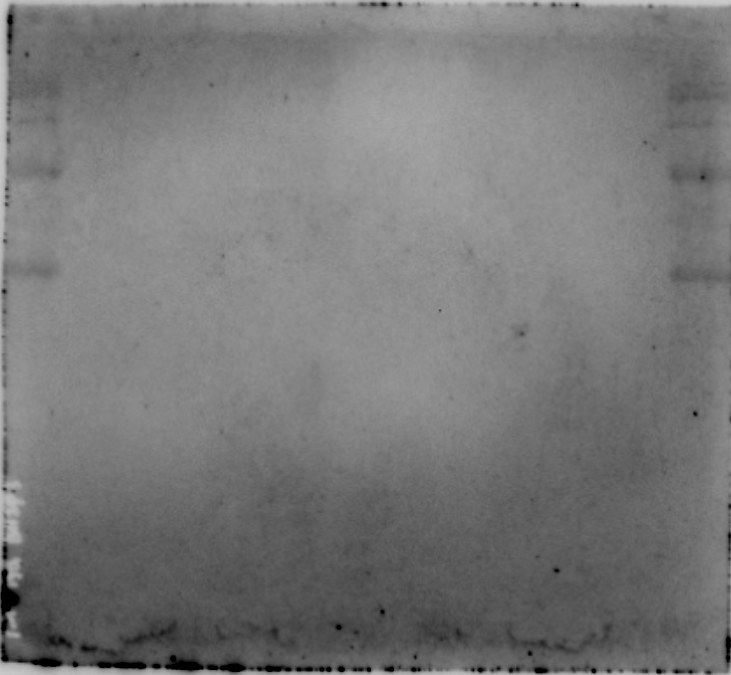

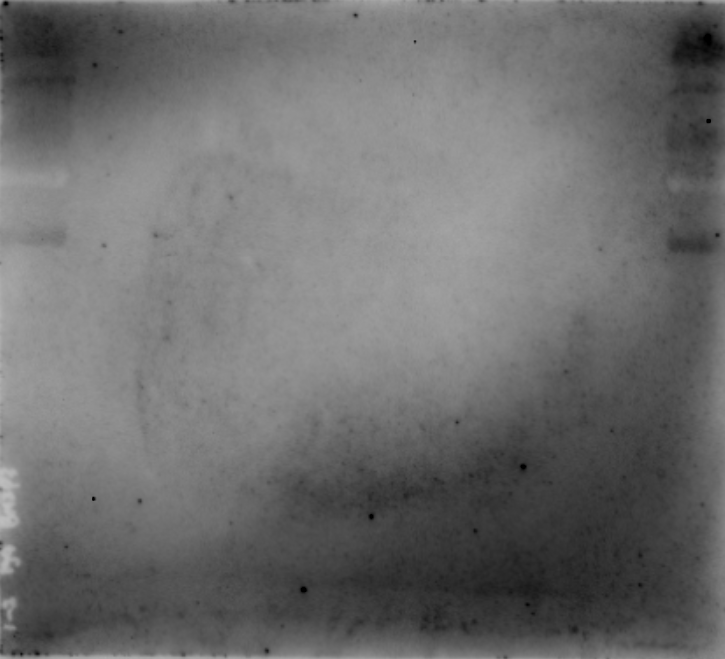

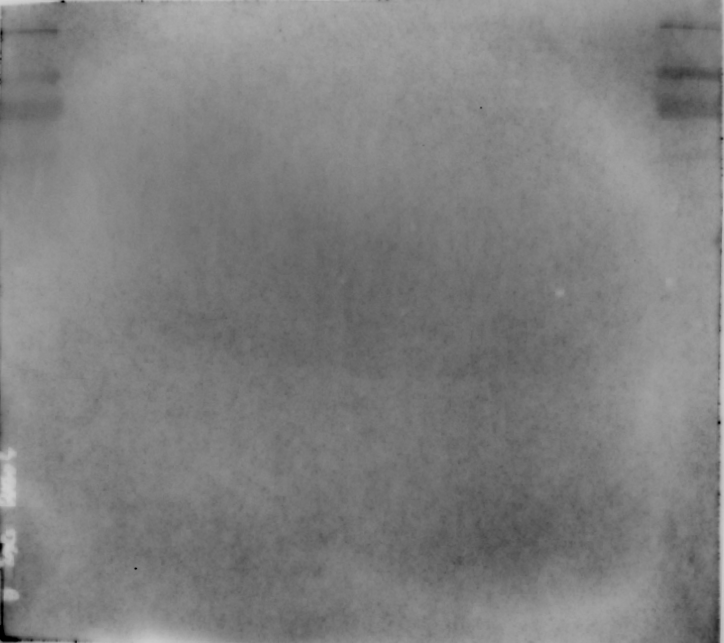


IP BCL2:BNIP3


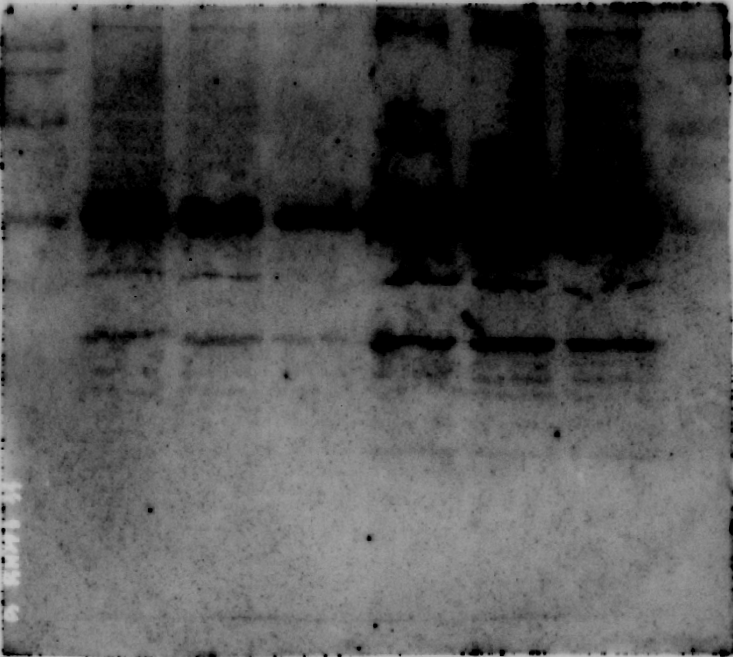

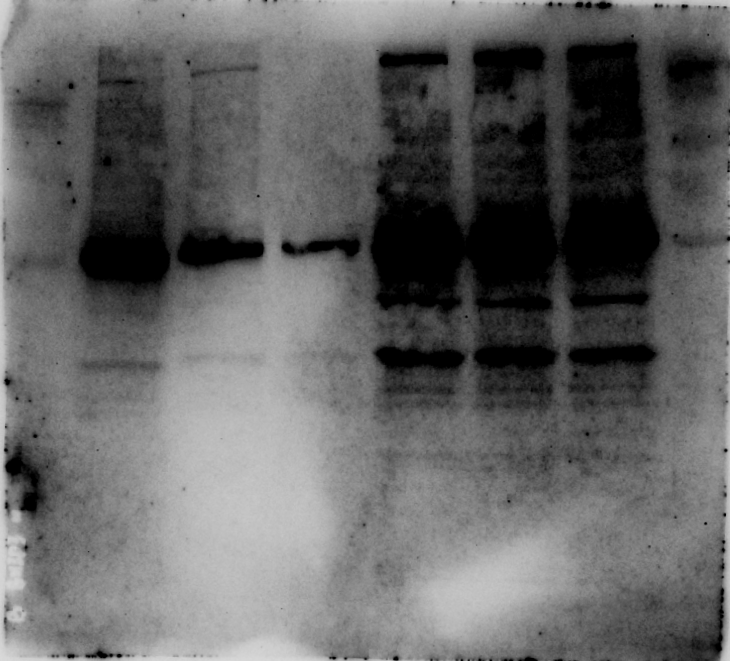

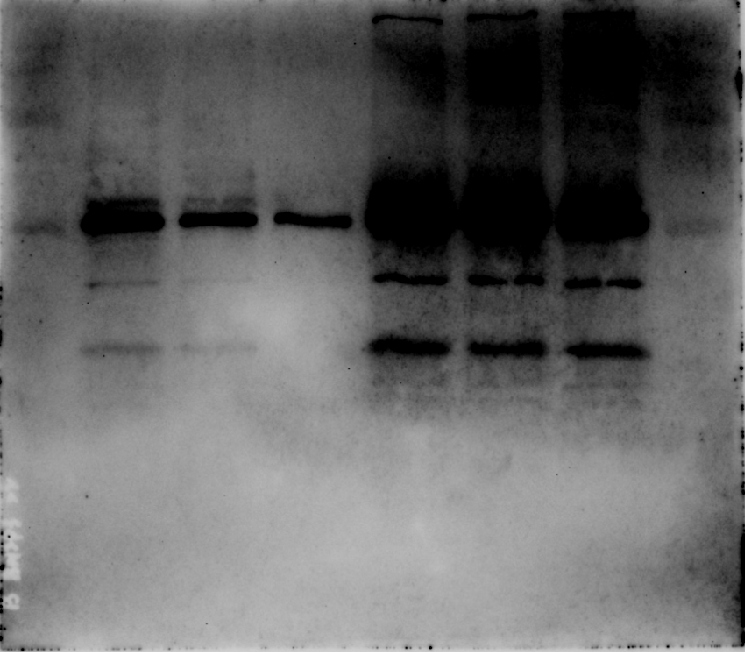


IP BCL2: BCL2


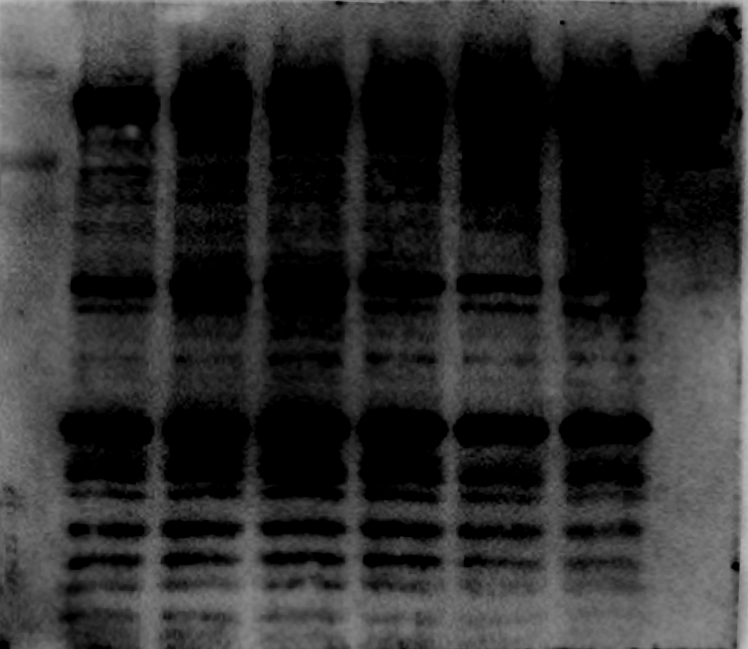

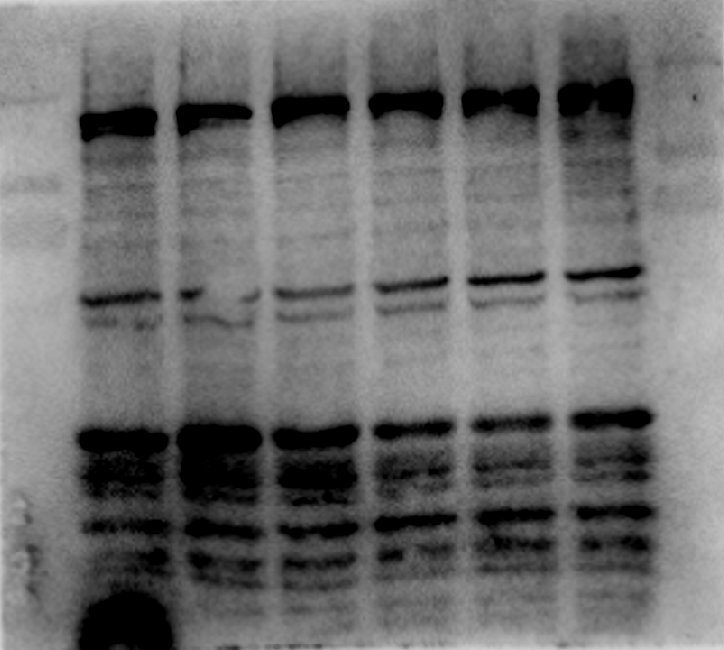

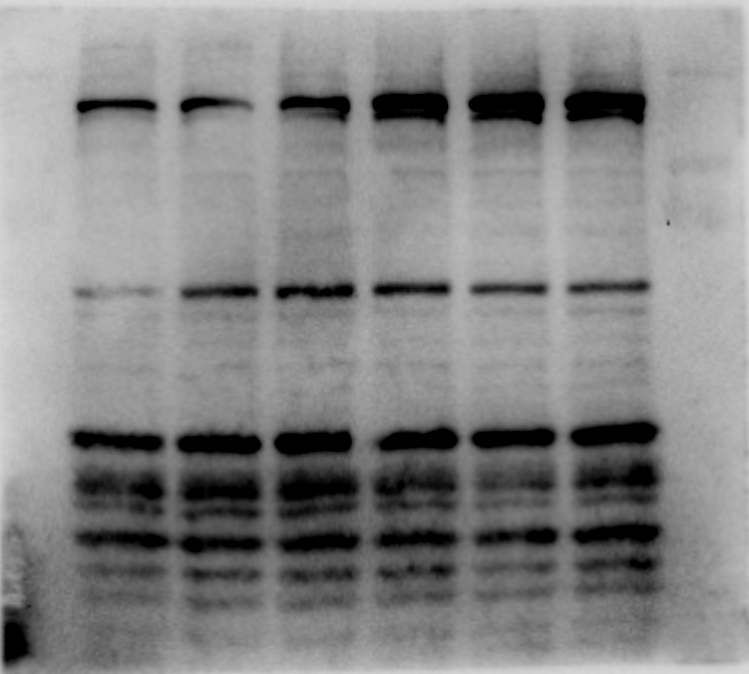


**Figure 6D：**

Input GAPDH


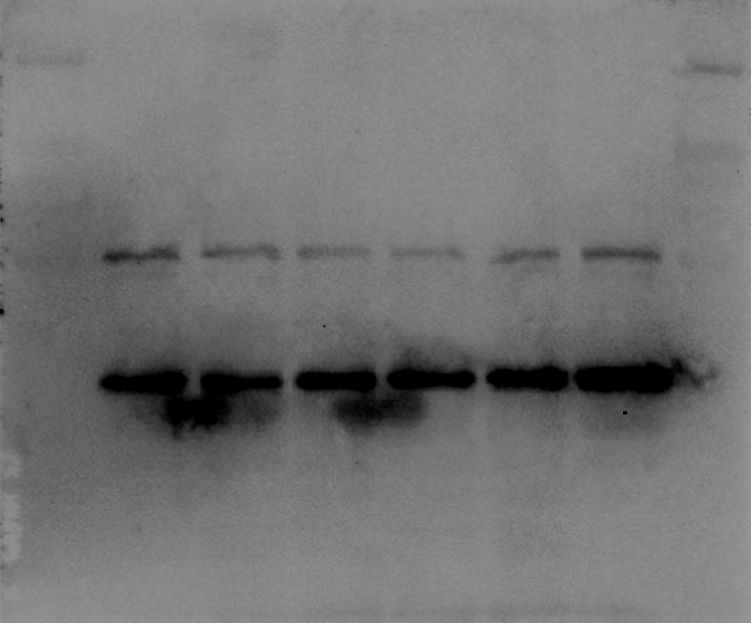

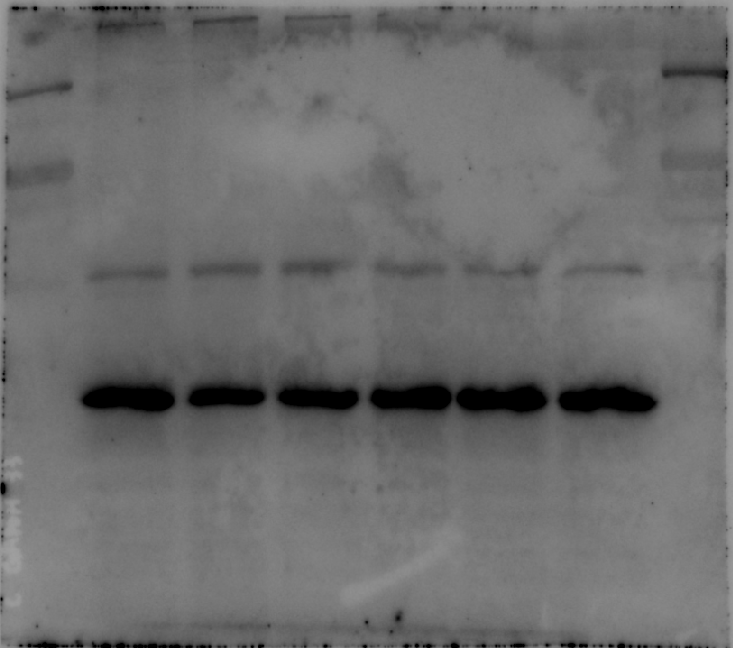

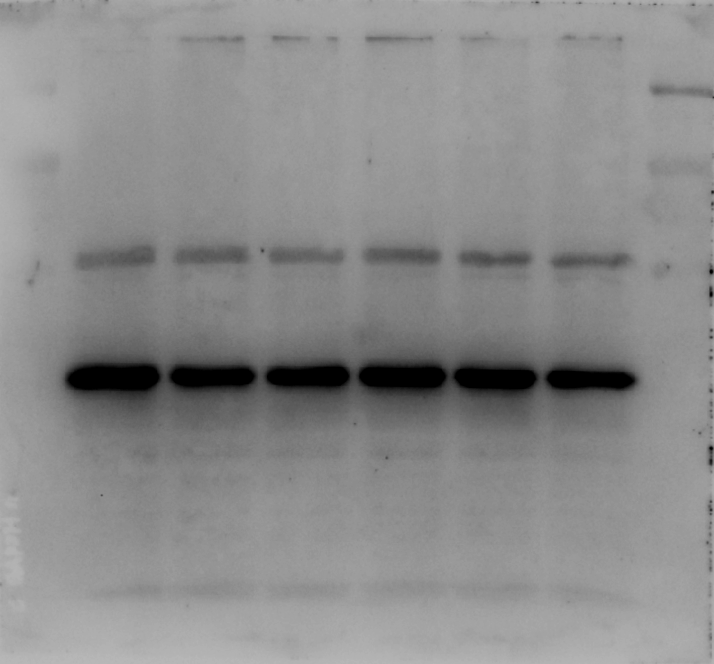


Input BECN1:


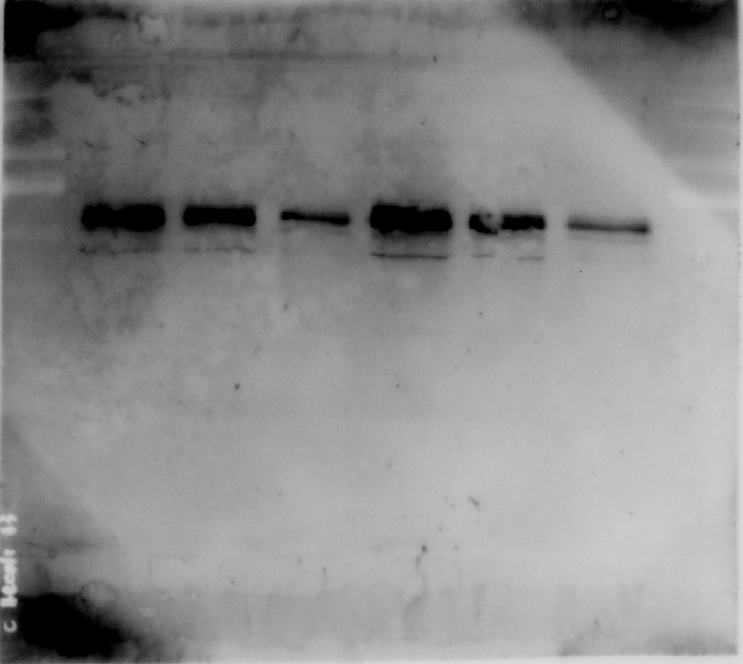

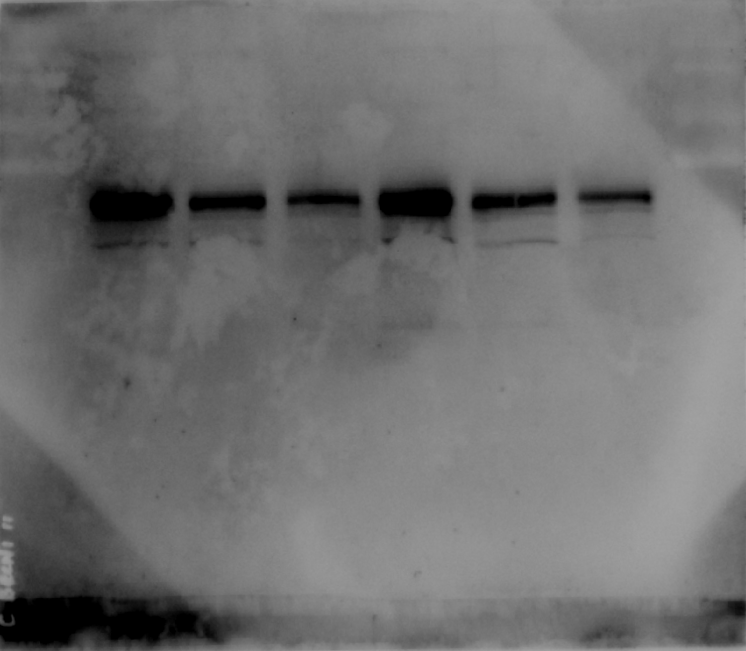

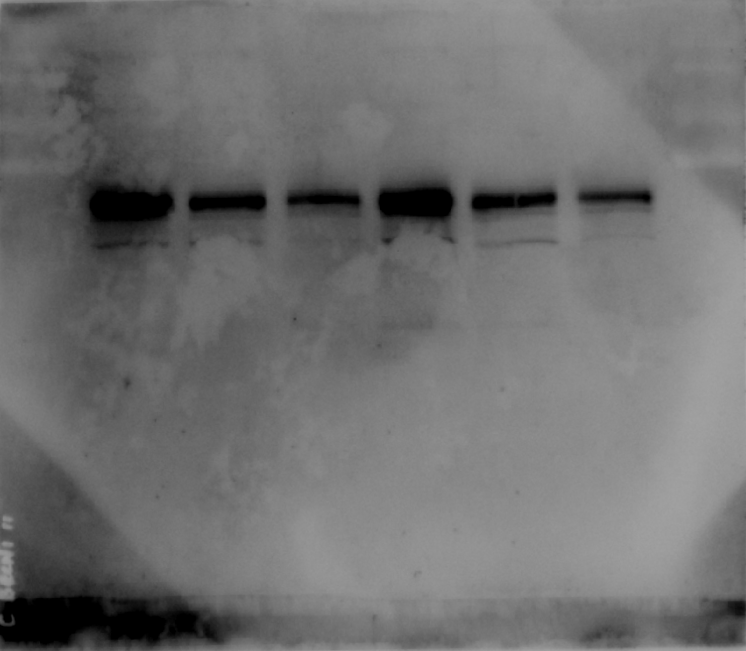


IP IgG：BECN1


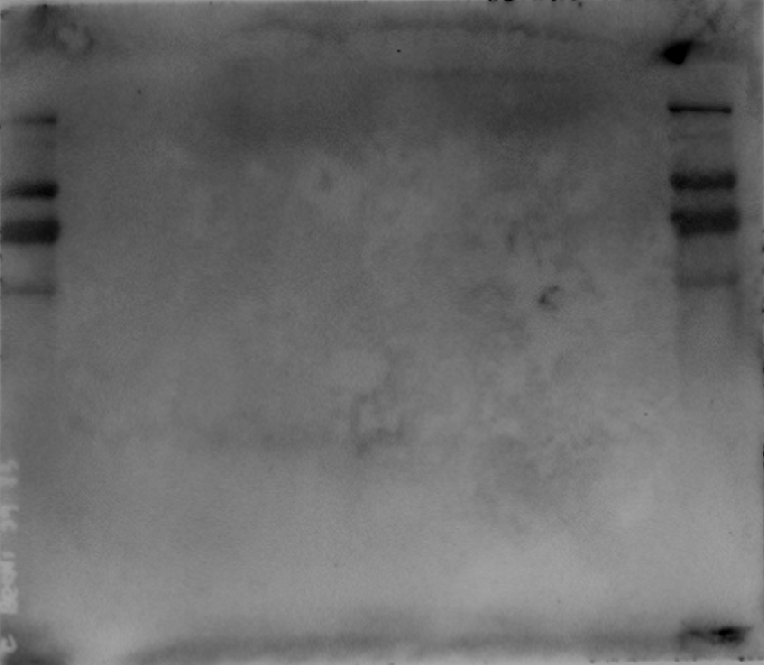

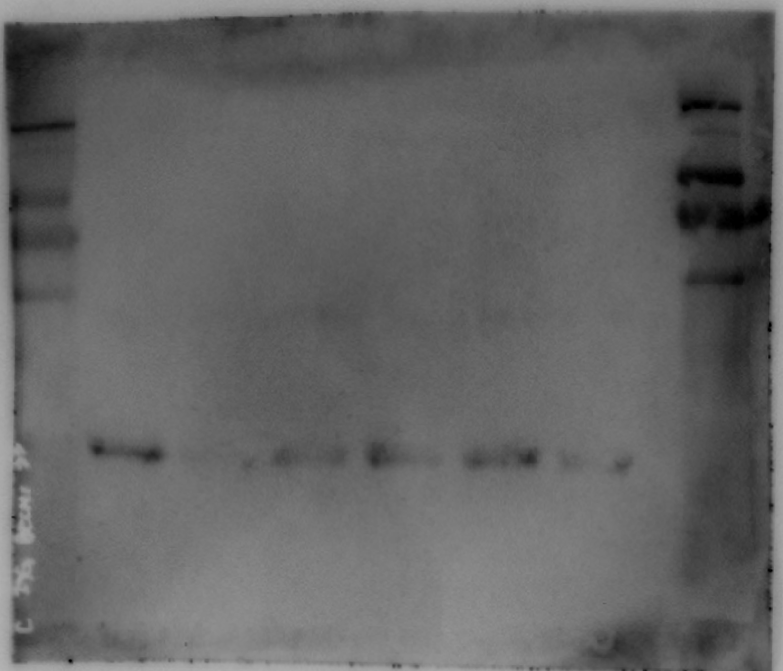

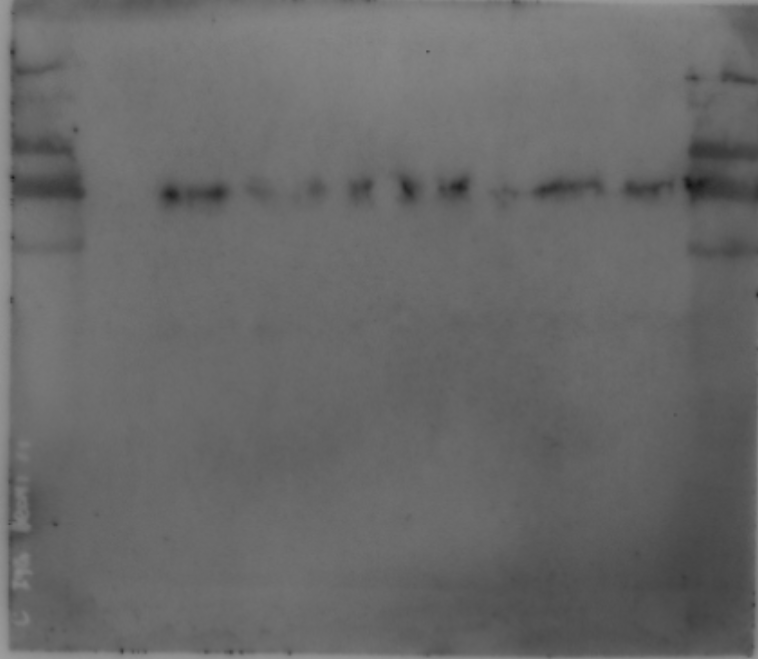


IP BCL2: BECN1


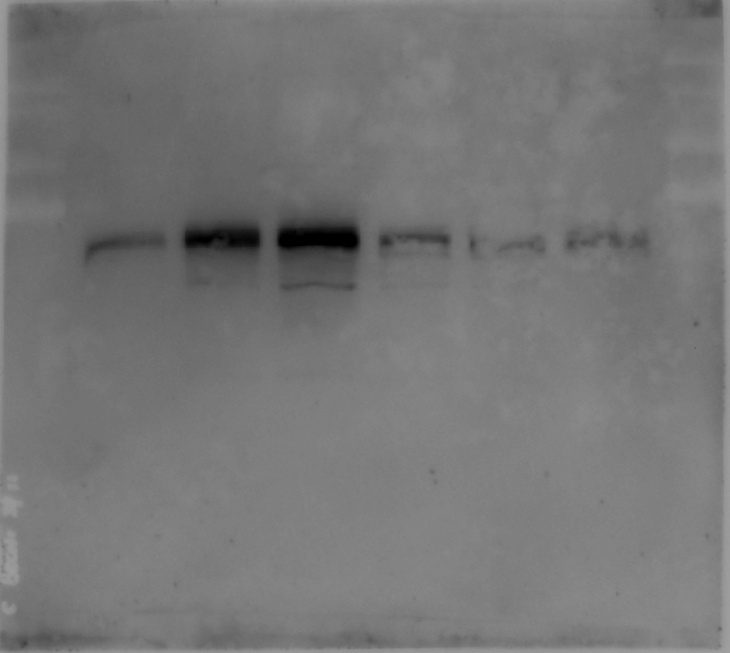

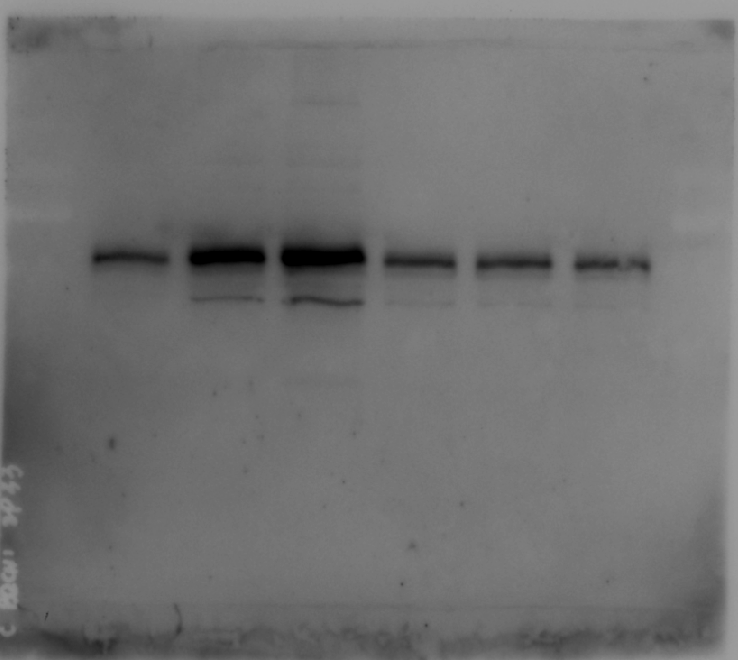

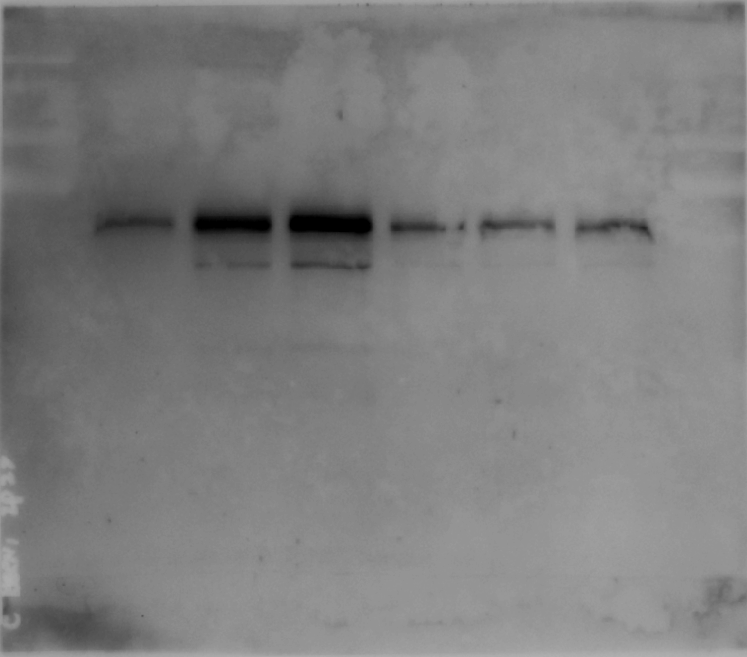


IP BCL2 BCL2


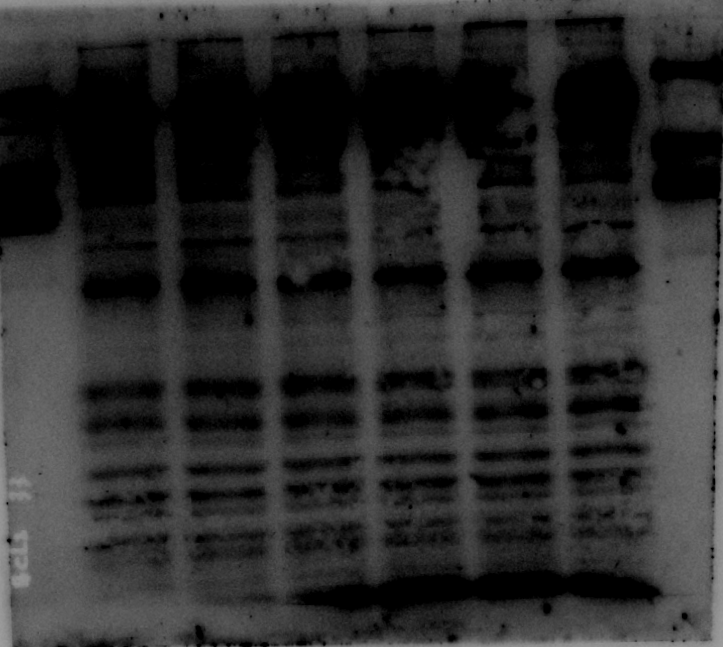

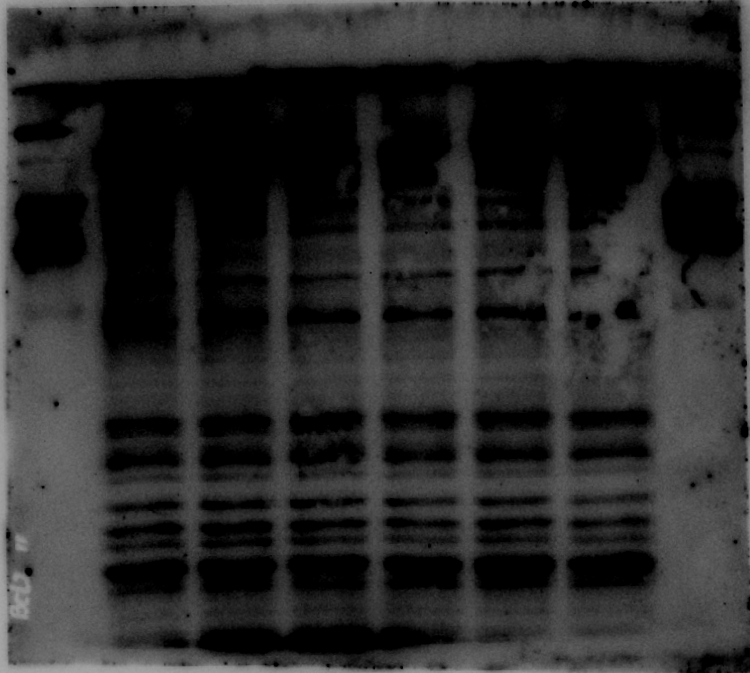

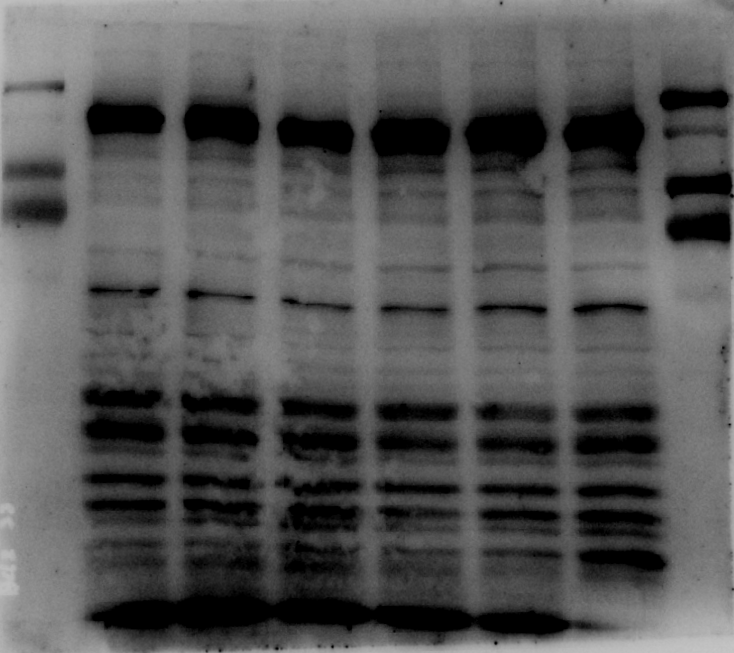


**Figure 6E:**

Input GAPDH


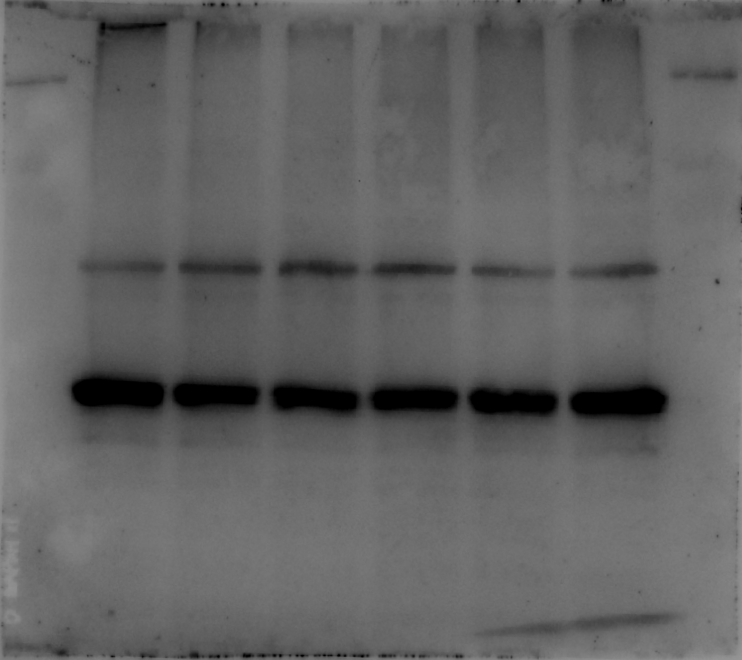

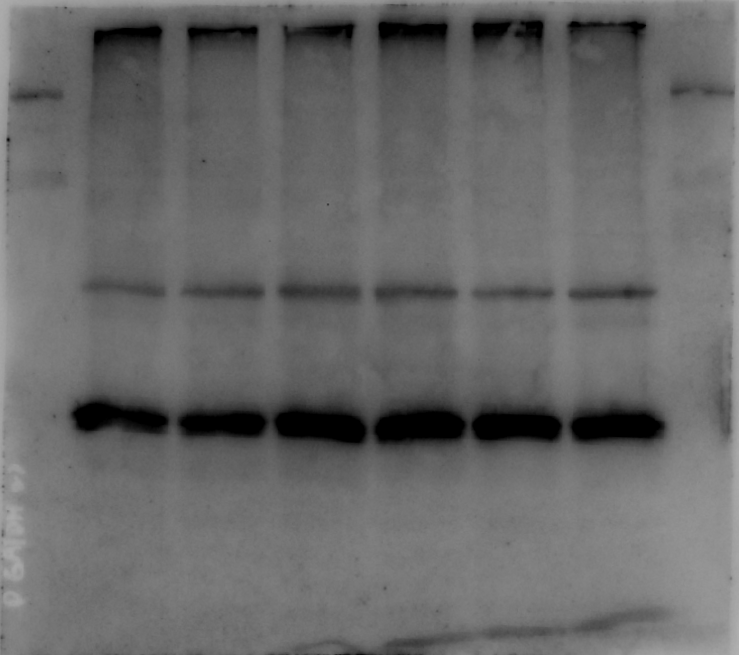

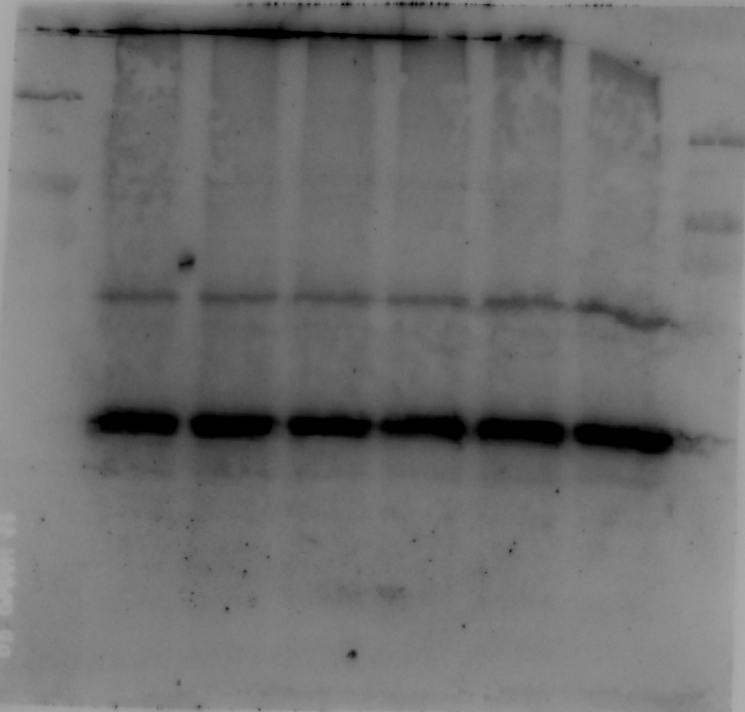


Input BCL2


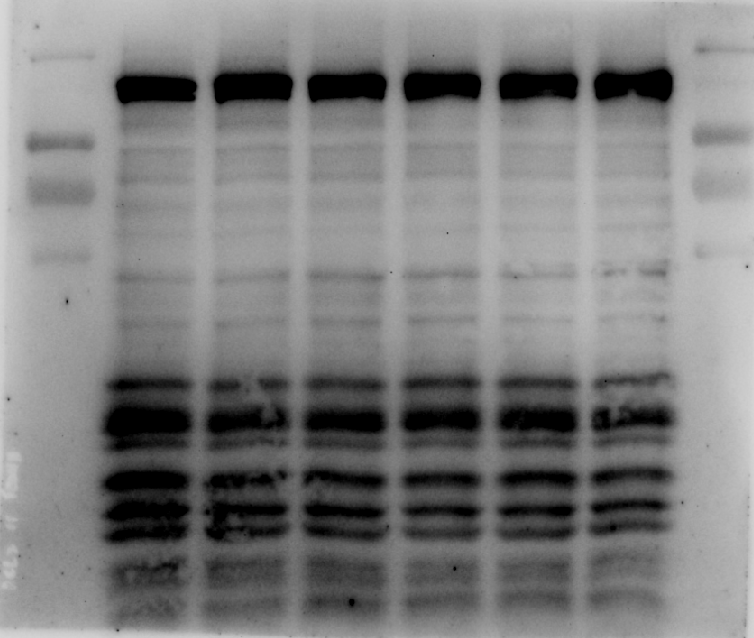

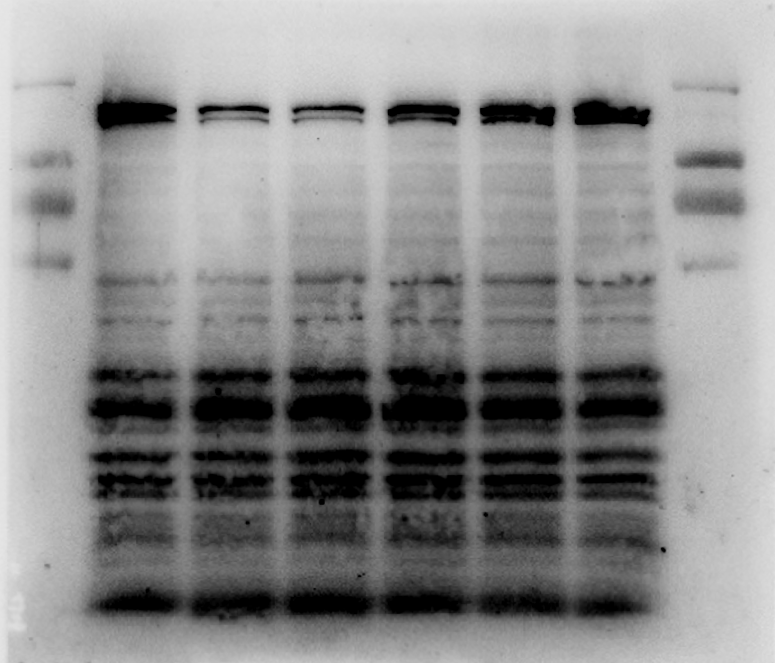

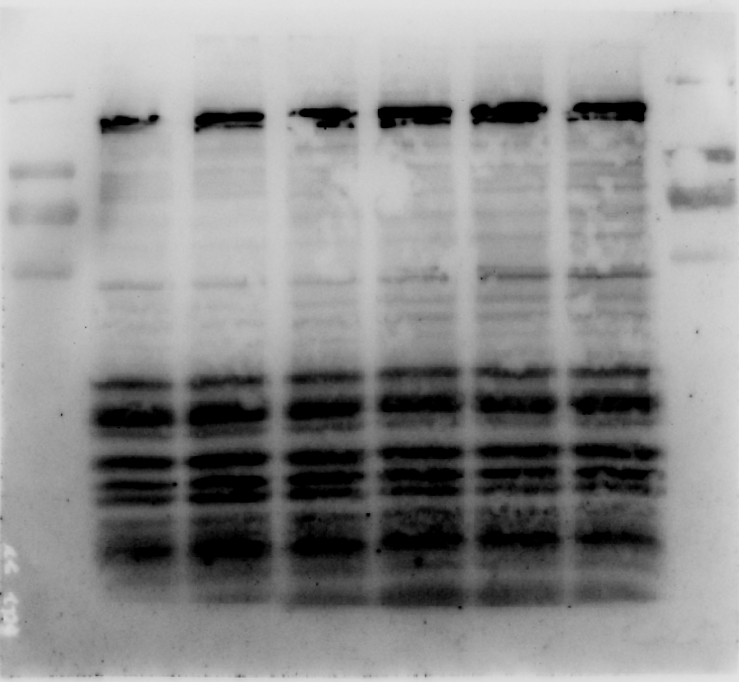


IP IgG：BCL2


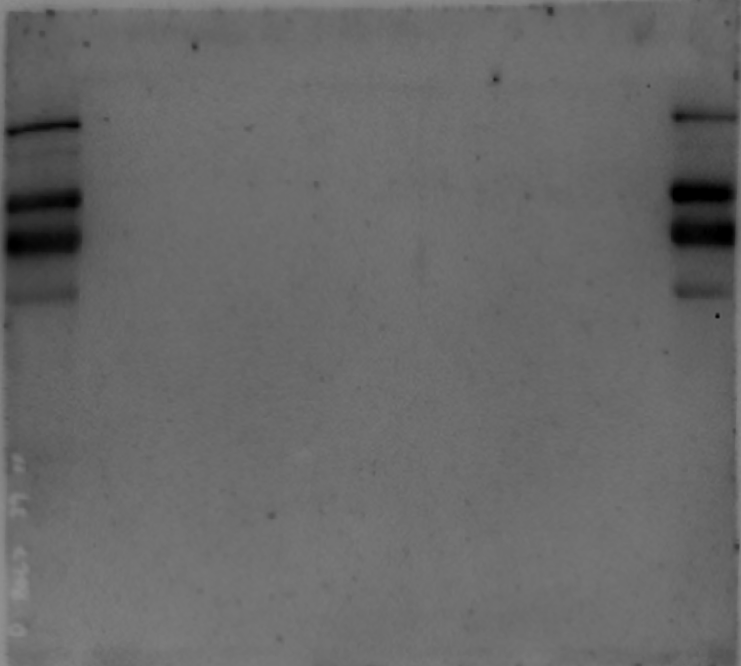

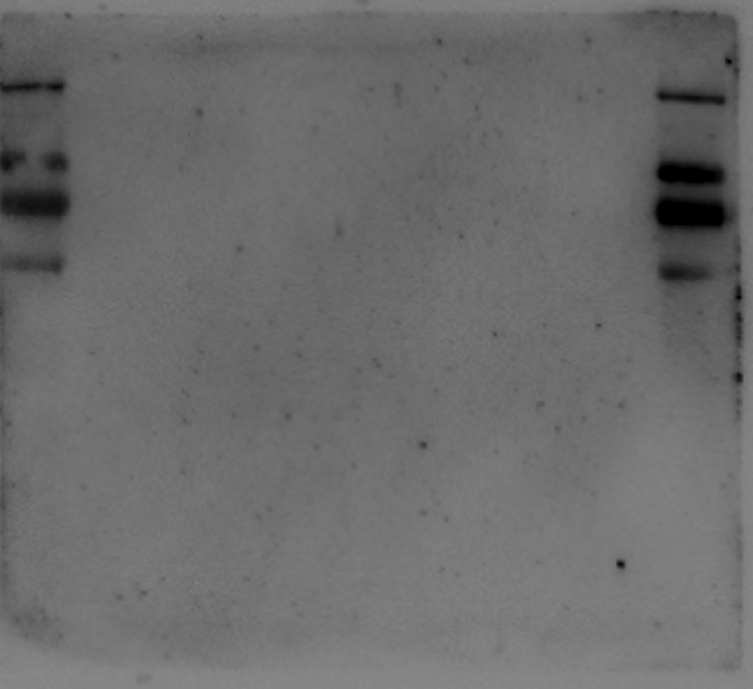

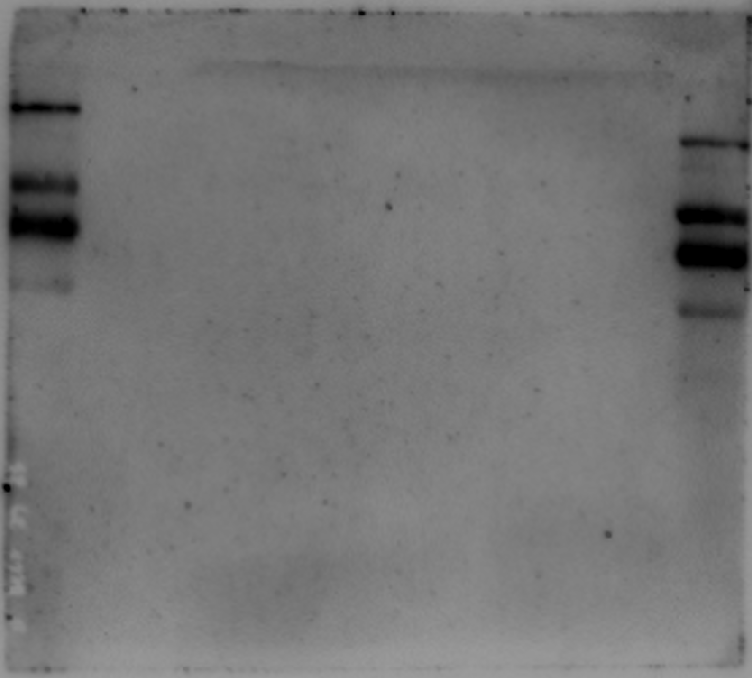


IP BECN1: BECN1


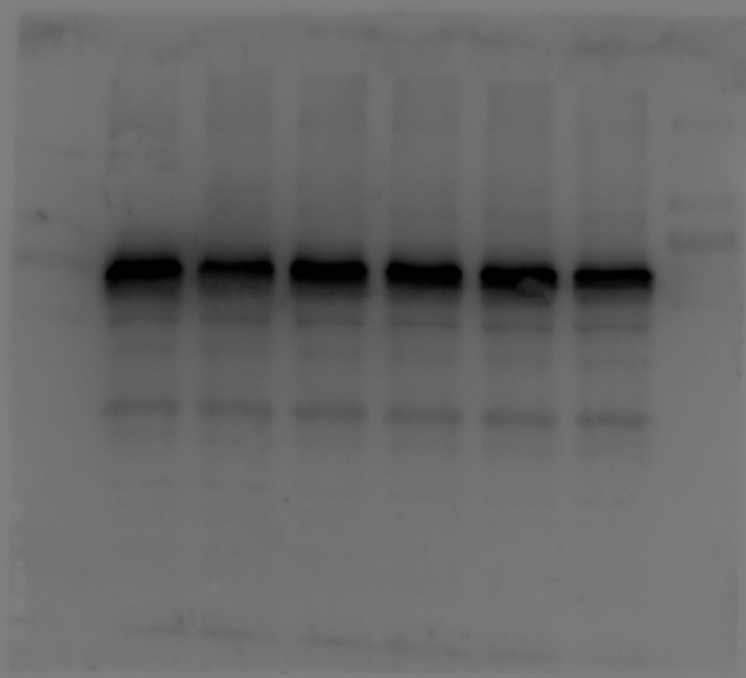

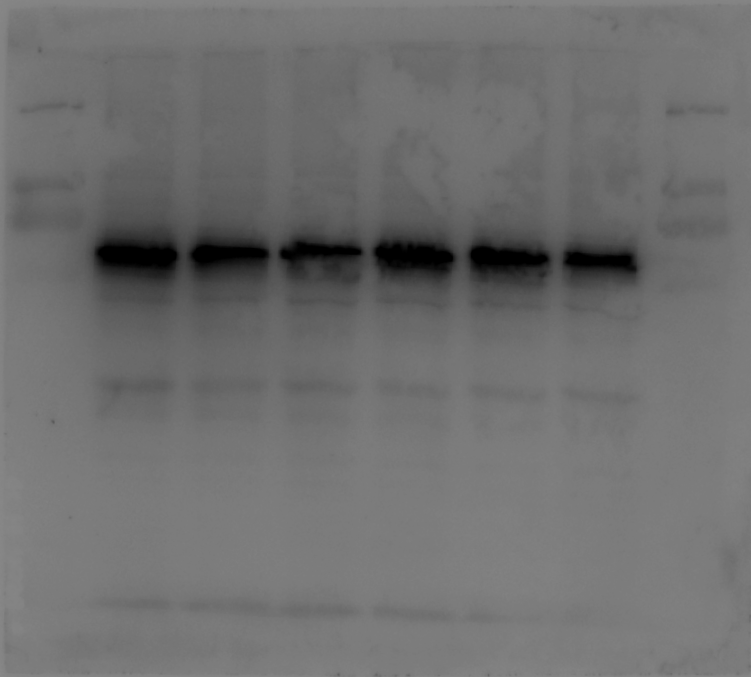

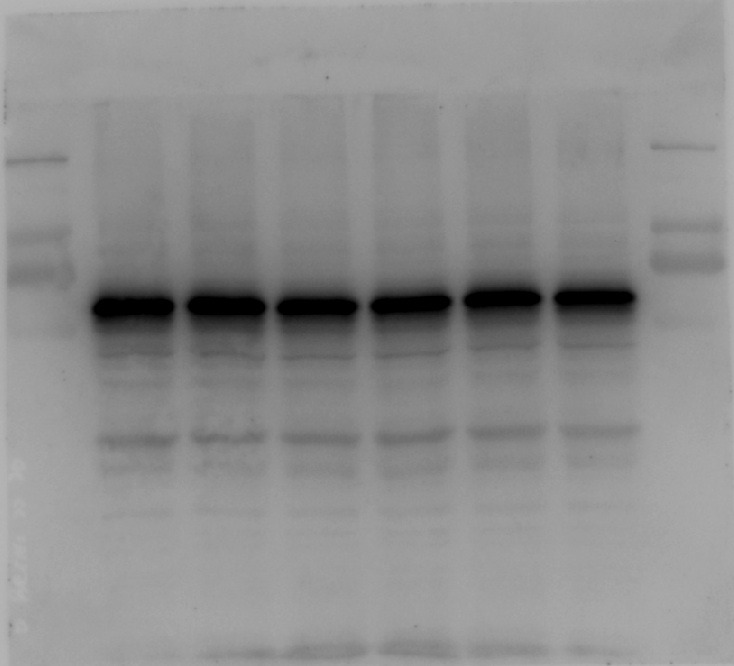


IP BECN1:BCL2


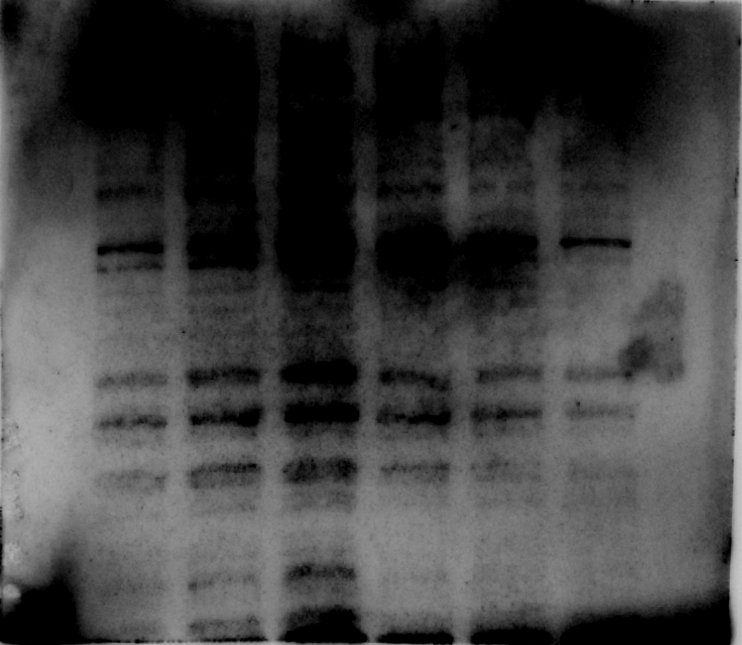

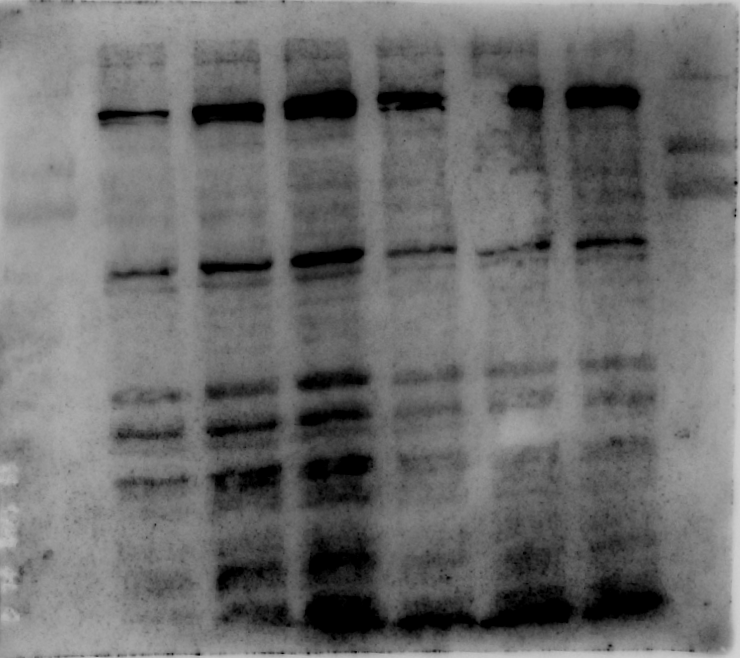

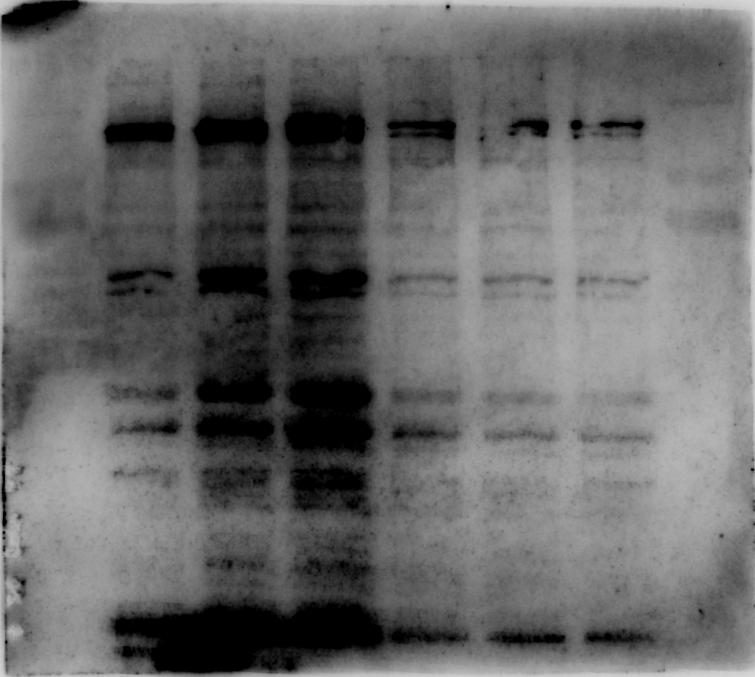


**Figure 7A:**

GAPDH


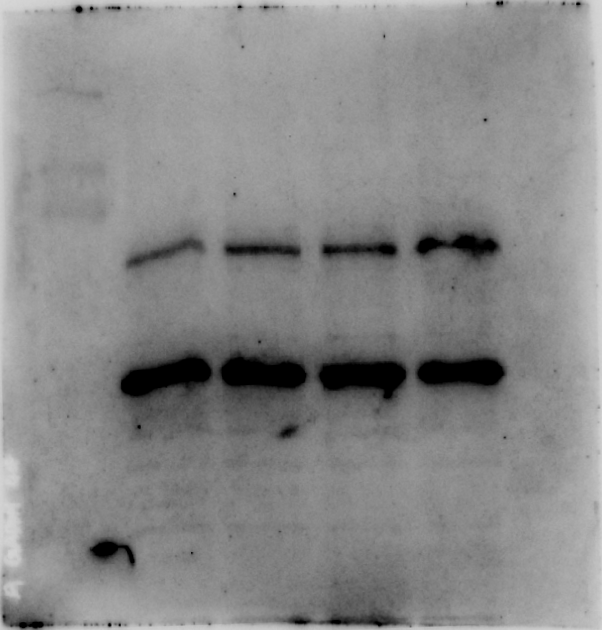

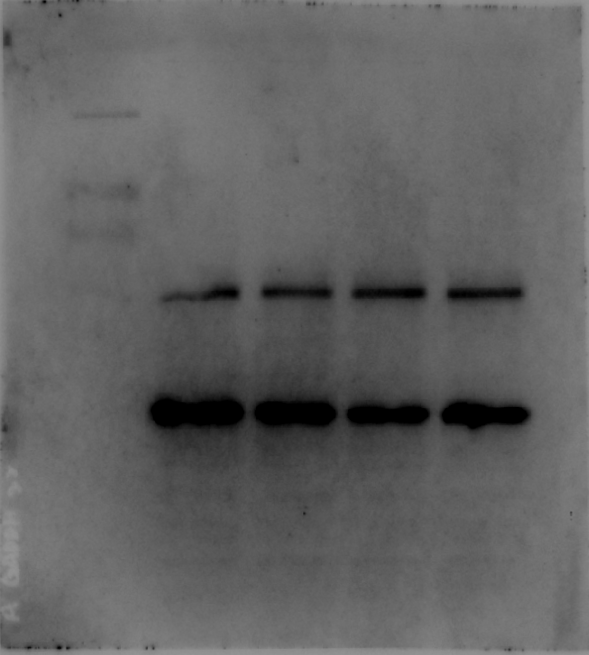

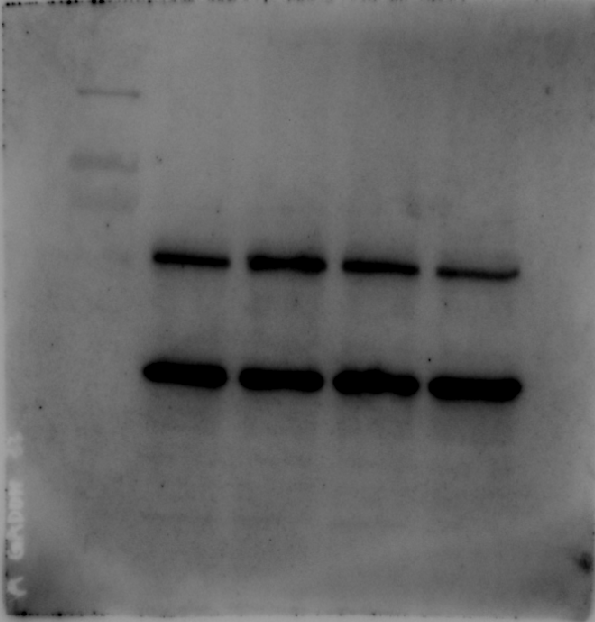


E1F1


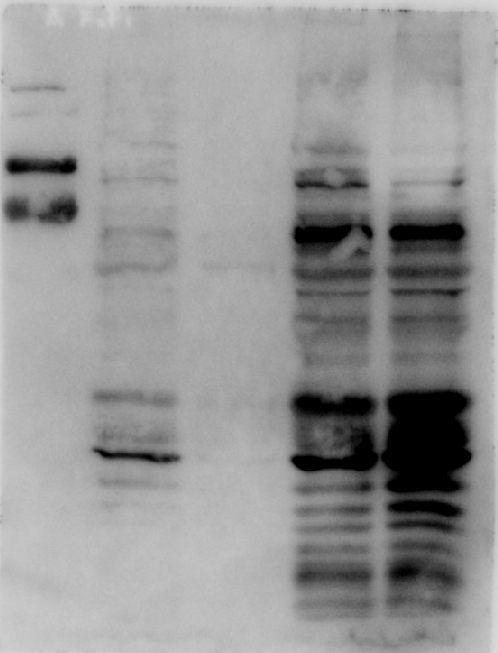

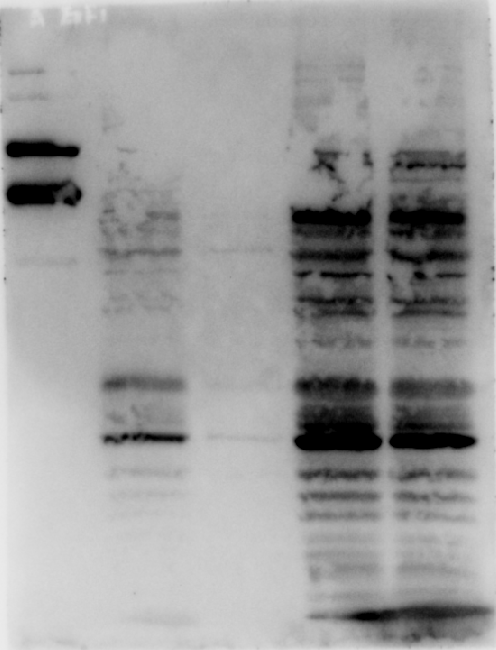

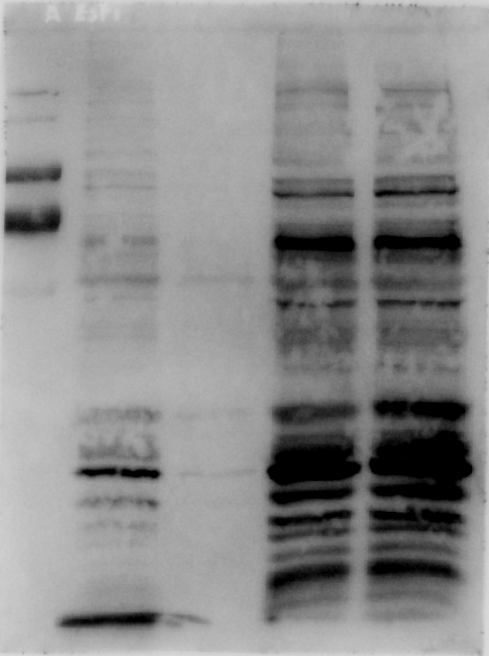


BNIP3


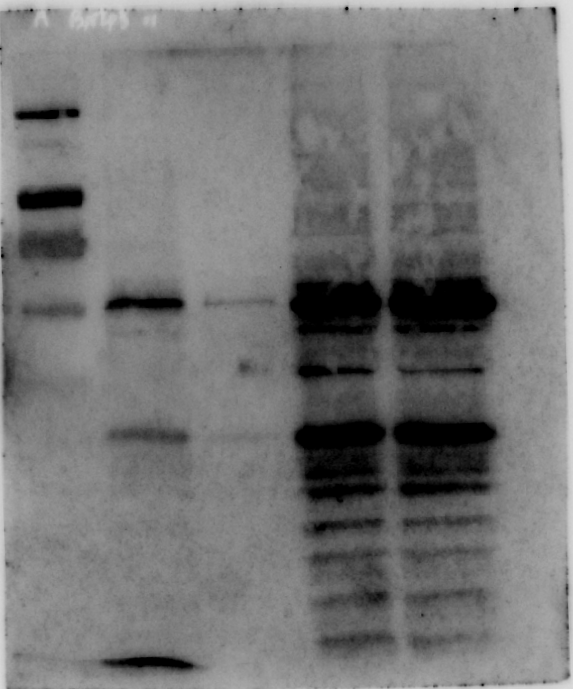

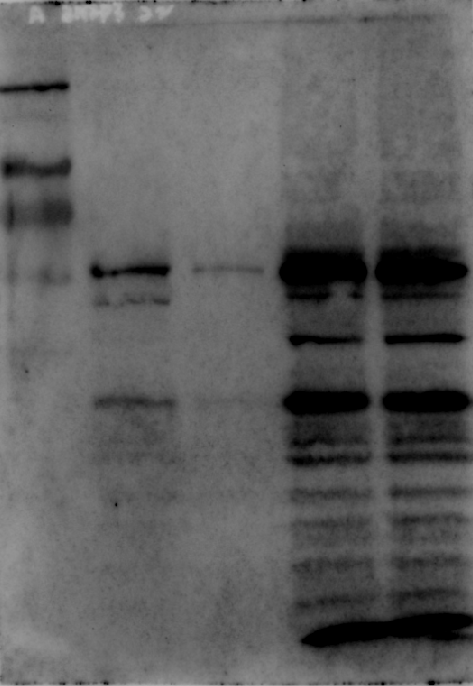

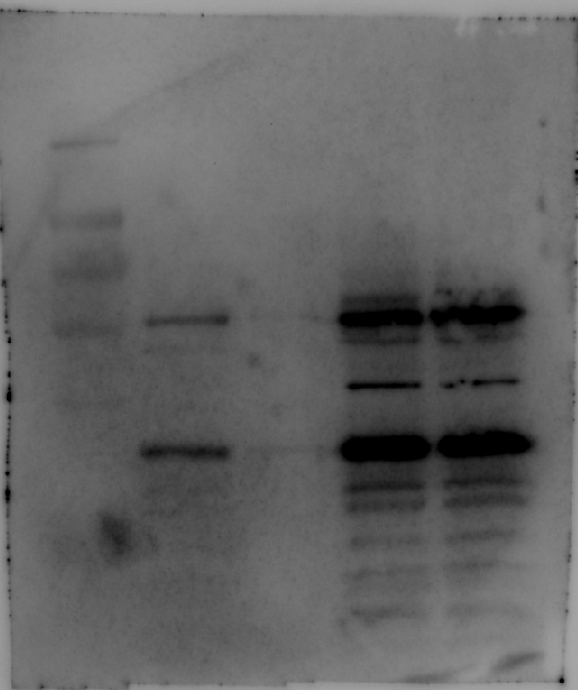


BECN1


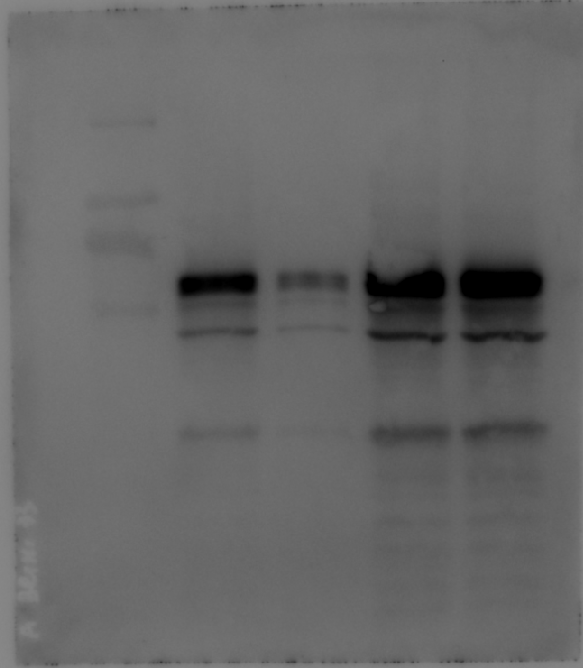

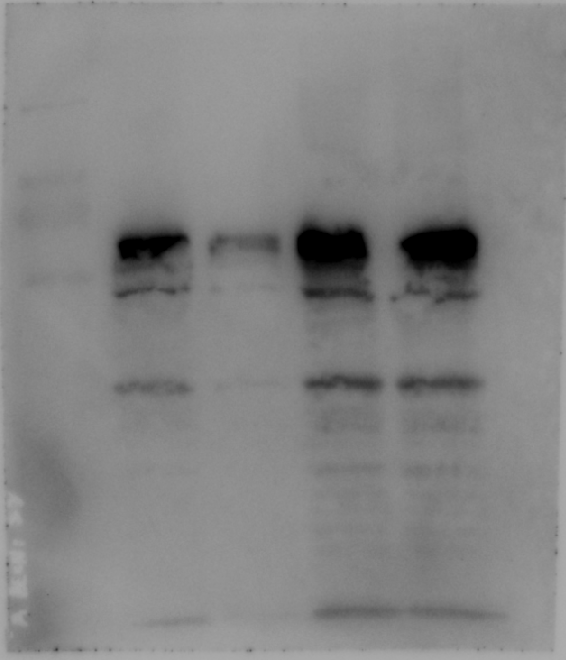

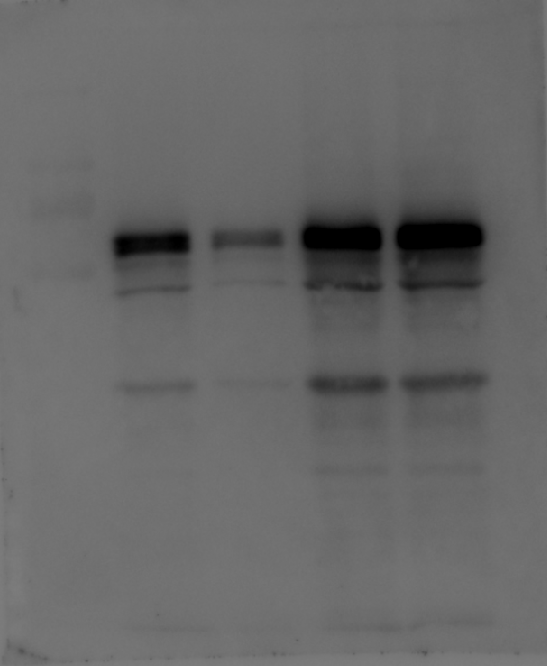


LC3


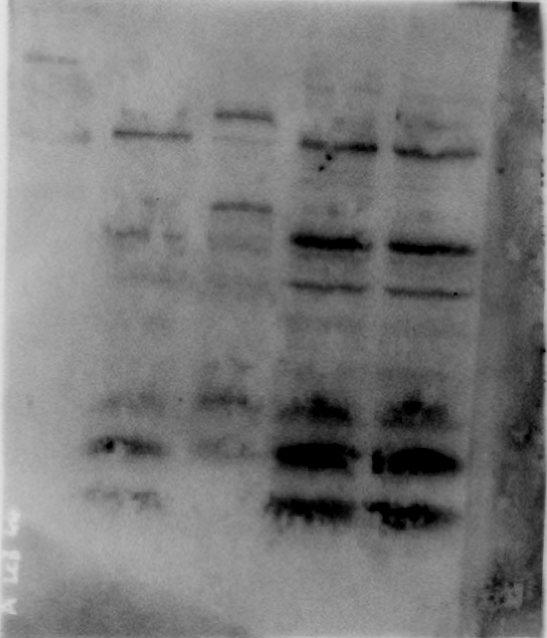

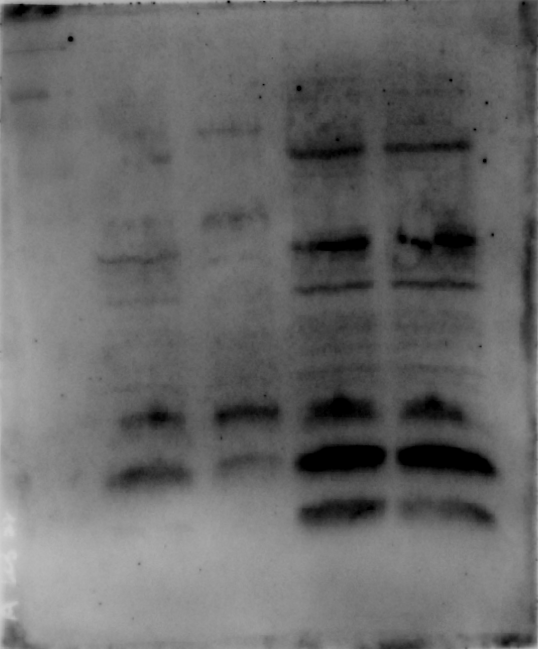

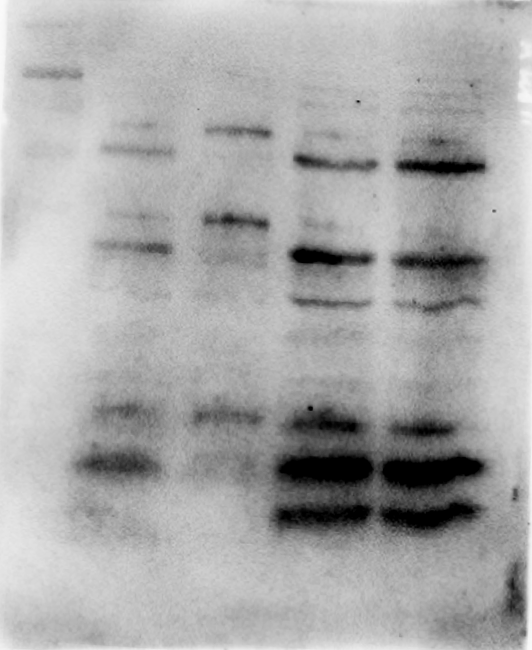

Supplement: Supplementary file 2 — Additional file 1. Supplementary material. [file 12964_2022_966_MOESM2_ESM.doc]
